# Cognitive Trajectories from Preclinical Alzheimer's Disease to Dementia

**DOI:** 10.1002/advs.202518124

**Published:** 2026-03-30

**Authors:** Fredrik Öhman, Lars Lau Raket, Michael Schöll, for the Alzheimer's Disease Neuroimaging Initiative†

**Affiliations:** ^1^ Department of Psychiatry and Neurochemistry Institute of Neuroscience and Physiology Sahlgrenska Academy University of Gothenburg Mölndal Sweden; ^2^ Wallenberg Centre for Molecular and Translational Medicine University of Gothenburg Gothenburg Sweden; ^3^ Department of Neuropsychiatry Sahlgrenska University Hospital Mölndal Sweden; ^4^ Clinical Memory Research Unit Lund University Malmö Sweden

**Keywords:** alzheimer's disease, biomarkers, clinical assessment, clinical trials, cognition, preclinical

## Abstract

Alzheimer's disease (AD) is marked by progressive cognitive decline, highlighting the importance of cognitive assessment in both clinical practice and research. This study investigates the temporal dynamics of cognitive decline and the differential sensitivity of cognitive measures across the AD spectrum. Cognitive measures, functional assessments, and amyloid‐beta (Aβ) and tau positron emission tomography (PET) scans from the Alzheimer's Disease Neuroimaging Initiative (ADNI) are analyzed using a latent‐time disease progression model. Data from 1,447 participants across cognitively normal, mild cognitive impairment, and dementia stages are positioned along an estimated AD timeline. The model estimates a ∼20‐year progression from initial Aβ PET positivity to late‐stage dementia. Cognitive trajectories reveal that cognitive measures deviate at different time points, with varying levels of abnormality across the disease continuum. These findings demonstrate that cognitive measures differ markedly in their sensitivity across the AD continuum. Characterizing when specific measures become abnormal provides a framework for stage‐appropriate test selection in clinical practice, improving clinical interpretation while informing the choice of cognitive endpoints and inclusion criteria in clinical trials.

## Graphical abstract

A continuous, multi‐domain characterization of cognitive decline across the Alzheimer's disease spectrum identifies when individual cognitive measures become abnormal. Episodic memory declines first, followed by executive function, language, processing speed, and visuospatial abilities, supporting improved clinical interpretation and optimized endpoint selection in clinical trials.

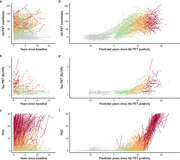

## 1 Introduction

Alzheimer's disease (AD) is a progressive neurodegenerative disorder characterized by the accumulation of amyloid‐β (Aβ) plaques and hyperphosphorylated tau tangles. These pathological changes begin long before clinical symptoms appear, progressing from a preclinical phase—characterized by pathology without apparent cognitive symptoms—to increasing cognitive and functional impairment [1]. Early symptoms typically involve deficits in episodic memory, followed by declines in executive function, attention, language, and visuospatial abilities [2]. These cognitive impairments vary across clinical stages [3], ultimately leading to dementia, which increasingly interferes with instrumental activities of daily living (IADL) as the disease progresses.

The significance of cognitive tests in clinical practice is well‐established [4], and as more effective treatments for AD become available [5], healthcare systems must be equipped to diagnose patients promptly to enable timely interventions. This growing need highlights the importance of selecting appropriate cognitive measures for accurate assessment. These cognitive measures are also important in clinical trials for investigational therapies [6]. While many trials increasingly target the early stages of AD, a reliance on tests with significant limitations, such as ceiling effects [7], persists. Therefore, prioritizing cognitive measures with proven validity [8, 9] is important for ensuring meaningful outcomes in clinical trials.

Although prior research has examined the sensitivity of cognitive tests at specific disease stages, a gap remains in continuously evaluating their performance across the full AD spectrum. To address this, we applied novel statistical methods for continuous‐time disease progression modeling [10, 11] to assess and compare cognitive decline across individual tests and composite measures, including screening assessments, from preclinical AD through mild cognitive impairment (MCI) to dementia. This approach enables characterization of the timing and sequence of cognitive changes across the AD continuum and comparison of the sensitivity of different measures at each stage.

## 2 Methods

### 2.1 The Alzheimer's Disease Neuroimaging Initiative (ADNI)

The data for this paper were sourced from the Alzheimer's Disease Neuroimaging Initiative (ADNI) database (https://adni.loni.usc.edu), launched in 2003. Led by Principal Investigator Michael W. Weiner, MD, ADNI was a collaborative effort aimed at determining whether serial magnetic resonance imaging (MRI), positron emission tomography (PET), along with other biological markers, and clinical and neuropsychological assessments can effectively track the progression of MCI and early AD.

The ADNI study received approval from the institutional review boards at each participating center, and all participants provided written informed consent (ClinicalTrials.gov registry numbers: ADNI GO: NCT01078636; ADNI 2: NCT0123197; ADNI 3: NCT02854033). The used dataset had a cut date of 16 January 2024.

#### 2.1.1 Participants

Participants included in this dataset were drawn from four ADNI cohorts (ADNI1, ADNI GO, ADNI2, and ADNI3) and were recruited at clinical sites throughout the United States and Canada. At enrollment, participants were clinically classified into baseline diagnostic groups—cognitively unimpaired (CN), MCI, or dementia—according to ADNI criteria [12]. We excluded patients without baseline disease severity assessment (*n* = 11), without a valid cerebrospinal fluid (CSF) or PET Aβ‐status at baseline (*n* = 456), who had non‐AD cognitive impairment, defined as a diagnosis of MCI or dementia or CDR global score greater than 0 or MMSE less than 26 without being Aβ+ (*n* = 515). The remaining 1,447 participants were classified into four groups according to their Aβ status and baseline clinical diagnosis: Aβ‐negative (Aβ‐) CN, Aβ‐positive (Aβ+) CN, Aβ+ MCI, and Aβ+ dementia.

#### 2.1.2 Cognitive Measures

In this study, we focused on cognitive measures that were widely used in both clinical and research settings and were readily available in the ADNI dataset, including cognitive screening instruments, individual neuropsychological tests, and cognitive composite measures. Screening measures included the Mini‐Mental State Examination (MMSE) [13] and the Montreal Cognitive Assessment (MoCA) [14], both widely used to assess global cognitive impairment.

Individual neuropsychological tests were used to assess specific cognitive domains. Memory was evaluated using the Rey Auditory Verbal Learning Test (RAVLT) [15], including the total score Across trials 1–5 (RAVLT immediate) and delayed 30‐min recall (RAVLT 30 min), as well as the Logical Memory (LM) subtest From the Wechsler Memory Scale (WMS) [16], assessing immediate and delayed narrative episodic memory. Attention and executive function were assessed using the Trail Making Test (Trails A and B) [17], the Digit Symbol Substitution Test (DSST) [18], and Digit Span tasks (forward and backward) [18]. Language abilities were measured using the Boston Naming Test (BNT30) [19] and semantic verbal fluency tasks (animals and vegetables) [20]. Visuospatial function was assessed using the Clock Drawing Test [21].

Cognitive composite measures included Alzheimer's Disease Assessment Scale–Cognitive Subscale (ADAS‐cog) [22], and a modified version of the Preclinical Alzheimer Cognitive Composite (mPACC) [23]. In the ADNI study, the modified PACC comprised MMSE, LM delayed recall, the delayed word recall from the ADAS‐cog, and either Trails B or DSST, depending on availability, as the original PACC variant, including the Free and Cued Selective Reminding Test (FCSRT), was not available in ADNI.

In addition, we used four domain‐specific cognitive composites—memory, executive function, language, and visuospatial abilities—derived from the ADNI cognitive battery using co‐calibration and psychometric harmonization methods [24]. These composites integrate multiple test items within each domain into standardized scores, enabling comparison across participants and accounting for differences in test versions and missing data.

#### 2.1.3 Functional Measures

The Functional Activities Questionnaire (FAQ), evaluating IADL, was used as an outcome measure in the disease progression model. Longitudinal analyses of FAQ scores were performed to investigate changes in functional status associated with AD over time.

#### 2.1.4 Biomarker Assessments

Biomarkers for the disease progression model were assessed using Aβ and tau PET imaging [25]. Aβ pathology was measured using florbetapir ([18F]AV‐45) [26], florbetaben ([18F]florbetaben) [27], or [11C]Pittsburgh Compound B, and quantified using centiloids [28] and standardized uptake value ratios (SUVRs). Tau tangle pathology was evaluated with [18F]flortaucipir and quantified in a previously established meta‐region of interest (ROI) using SUVR [29]. All imaging data were processed according to ADNI standards, including automated quality assurance metrics and visual inspection.

Aβ status (Aβ+/Aβ−) was determined using CSF or PET at every visit where either was available. For CSF, Aβ‐positivity was assessed via the Aβ42/40 ratio, measured either on a cobas e 601 analyzer using Roche Elecsys immunoassays (positivity cutoff <0.066) or by 2D‐UPLC tandem mass spectrometry (positivity cutoff <0.138) [30]. PET tracers [18F]florbetapir, [18F]florbetaben, and [11C]Pittsburgh Compound B were used to establish Aβ‐PET positivity using published ADNI PET core cutoffs, defined by SUVR in a composite cortical region referenced to the cerebellum (Florbetapir >1.11; Florbetaben >1.08; Pittsburgh Compound B >1.22) [25, 26, 27]. Negative Aβ status was carried backward when baseline measures were missing, and positive status was carried forward. For discrepant CSF and PET results, cognitively unimpaired subjects were considered Aβ+ if either biomarker was abnormal, whereas cognitively impaired subjects were considered Aβ+ only if PET was positive.

### 2.2 Statistical Methods

#### 2.2.1 Disease Progression Model

To derive a continuous staging of patients along a continuum, we used a statistical disease progression model allowing prediction of an individual's disease stage based on Aβ PET, tau PET, and FAQ [10, 11], using either single or longitudinal measurements.

As outcome measures, we used observations of Aβ and tau PET and the FAQ. Let *y_ijk_* denote subject *i*’s observation of the *k*th outcome measure (*k = 1, 2, 3*), *t_ij_* years after the baseline visit. The mean trajectory θ_*k*_ of the *k*th outcome over the assumed disease continuum was estimated from the model.

(1) $$y_{ijk}=\theta_{k}\;\left(t_{ij}+s_{\mathrm{bl}\;\mathrm{status}\left(i\right)}+s_{\mathrm{bl}\;A\beta -\left(i\right)}+s_{i}\right)+x_{ik}+e_{ijk}$$

where *t_ij_* + *s*_bl status(*i*)_ + *s*_bl Aβ‐(*i*)_ + *s_i_* is the time argument that is shared across outcomes, which we will refer to as *disease time*.

The model parameters were modeled as follows:

- θ_*k*_ was a monotone Hermite spline with 5 degrees of freedom. Knots were placed relative to the average predicted disease time of the CN Aβ+ group at baseline, with knots placed at ‐8.33, 0, 8.33, 16.67, and 25 years, and an additional two knots replicating the boundary values placed 1 month before and after these respective knots to limit boundary artifacts. This configuration reflects the principle that spline estimates were generally robust to knot placement within a reasonable range; however, too few knots may result in oversmoothing, whereas excessive knots increase the risk of overfitting and spurious oscillations [31].
- *s*_bl status(*i*)_ was a fixed effect time‐shift describing the average shift in disease time of the baseline status group (cognitively unimpaired, subjective memory complaint, early MCI, late MCI, or dementia) of subject *i*.
- *s*_bl Aβ‐(*i*)_ was a fixed effect time‐shift describing the average shift in disease time associated with Aβ‐ status at baseline.
- *s_i_* was a random effect time‐shift describing the time deviation of subject *i* relative to their baseline group and Aβ+/Aβ‐ status.
- *x_ik_* was a random effect intercept describing subject *i*’s consistent vertical deviation in outcome measure *k*, and an unstructured covariance matrix was used to model the within‐subject correlation across outcomes.
- *e_ijk_* was independent identically distributed Gaussian noise with separate variance parameters for each outcome *k*.

**FIGURE 1:**
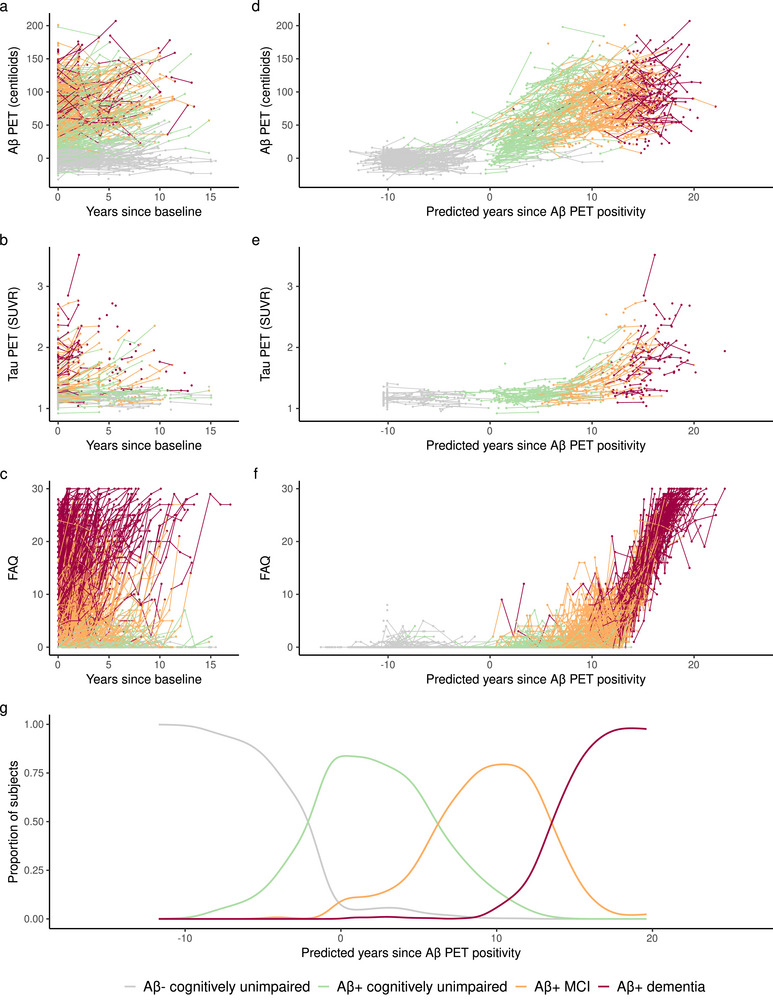
Visualization of observed data (a–c), observed data on synchronized time scale estimated by disease progression model (d–f), and the distribution of diagnoses across the estimated disease continuum based on Aβ status (g). Line plots depicting the alignment of observed samples from specific subjects to the left, and the data synchronized by the disease progression model to the right. Individual subjects are shown as separate lines with colors showing Aβ status and clinical diagnosis at each visit. Abbreviations: Aβ, amyloid beta; PET, positron emission tomography; SUVR, standardized uptake value ratio.

To avoid overparameterization, the disease time scale was anchored by parameterizing the fixed effect time shifts such that a disease time of 0 corresponded to the time at which a subject, on average, reached Aβ+ status measured by PET with a threshold of >26 CL. The predicted disease time thus represents predicted years since Aβ PET positivity. The estimation process is illustrated in Figure 1. All parameters in the model were estimated using maximum likelihood under a missing‐at‐random assumption. No imputation was performed. Maximum a posteriori estimation was used for the prediction of random effects.

The analyses were conducted using R (version 4.2.3) and the progmod R package [32].

#### 2.2.2 Analysis of Cognitive Test Results

Cognitive scores can be influenced by factors unrelated to AD, such as age, sex, and education [33]. Adjusting for these factors can improve measurement precision [34]. In this study, we derived *T*‐scores for each observed cognitive test outcome that reflected the normalized deviation of the test result compared to an Aβ‐, CN individual with the same age, education, and sex. The reference results were derived by fitting a linear mixed‐effects model with a subject‐level intercept that adjusted the results of each cognitive test for age, education, and sex in the reference population consisting of participants who were Aβ‐ CN. T‐scores were computed by subtracting the estimated age, education, and sex effects for each individual's test results, and normalizing the residual by the standard deviation (SD) in the reference population.

Trajectories of *T*‐scores of cognitive tests were analyzed along the estimated disease continuum described above, where each patient had their disease stage at each visit was predicted. Trajectories were modeled using a mixed‐effects model with the mean over the predicted disease time modeled as a natural cubic spline and a random subject‐level intercept. The degrees of freedom (0–8) of the spline trajectory were determined using the Bayesian Information Criterion.

To assess associations between cognitive trajectories and predicted disease time, Spearman rank correlations were computed across all observations. To visualize cognitive performance across disease stages, we plotted each cognitive measure against predicted years since Aβ PET positivity. Quantile regression was used to estimate the 25th, 50th, and 75th percentile trajectories, providing a distribution‐free characterization of cognitive scores at each disease stage. For each measure, we fit cross‐sectional quantile regression models using natural cubic splines with degrees of freedom ranging from 0 (intercept only) to 15. The optimal spline complexity was selected using the Bayesian Information Criterion (BIC). Curves were estimated over the fifth to 95th percentile range of observed disease time values to avoid extrapolation. Quantile regression was implemented using the lqm function from the lqmm R package [35].

### 2.3 Data Availability 

Data used in this study were obtained from the ADNI database (https://adni.loni.usc.edu). ADNI data were publicly available to qualified researchers upon registration and approval through the ADNI Data Use Agreement.

## 3 Results

### 3.1 Sample Characteristics and Group Differences

Table 1 presents the baseline characteristics of the four biomarker‐stratified groups: Aβ‐ CN (*n* = 384), Aβ+ CN (*n* = 293), Aβ+ MCI (*n* = 500), and Aβ+ dementia (*n* = 270). Age increased progressively across groups, ranging from 70.2 (Aβ‐ CN) to 74.4 years (Aβ+ dementia). Education levels were consistent across groups, with a median of 16 to 17 years. The proportion of females ranged from 56.5% to 60.8% in CN groups, compared to 42.4% to 44.8% in the MCI and dementia groups.

**TABLE 1:** Sample characteristics. Numbers are presented as mean and standard deviation, except age, education, follow‐up time, and number of visits, which are presented as median and interquartile range. Abbreviations: Aβ, amyloid; Aβ‐, amyloid negative; Aβ+, amyloid positive; ADAS‐cog, Alzheimer's Disease Assessment Scale; DSST, Digit symbol substitution test; MCI, Mild Cognitive Impairment; mPACC, The Preclinical Alzheimer Cognitive Composite; MMSE, Mini‐Mental State Examination; MoCA, Montreal Cognitive Assessment; PET, positron emission tomography; RAVLT, Rey Auditory Verbal Learning Test. *The scale direction is inverted, meaning higher values indicate worse performance.

Baseline sample	Aβ‐ CN *n* = 384	Aβ+ CN *n* = 293	Aβ+ MCI *n* = 500	Aβ+ dementia *n* = 270
Age	70.2 [66.4,74.6]	73.4 [68.9, 78.1]	73.9 [68.7, 78.2]	74.4 [69.1, 79.6]
Education	17 [15, 18]	16 [15, 18]	16 [14, 18]	16 [13, 18]
Female	217 (56.5%)	178 (60.8%)	212 (42.4%)	121 (44.8%)
Follow‐up time (years)	4.0 [2.0, 7.1]	4.0 [2.2, 6.4]	3.2 [2.0, 5.1]	2.0 [1.0, 2.1]
Number of visits	5 [3, 8]	5 [3, 8]	7 [4, 9]	4 [3, 5]
Aβ PET Centiloids	0.0 (8.64)	45.6 (37.0)	75.6 (35.4)	92.4 (33.7)
Tau PET Meta‐temporal SUVR	1.17 (0.07)	1.24 (0.17)	1.52 (0.38)	1.71 (0.37)
FAQ	0.1 (0.71)	0.2 (0.59)	3.7 (4.26)	13.1 (6.77)

Cognitive measures				
MMSE	29.2 (1.05)	29.1 (1.08)	27.3 (1.92)	23.1 (2.20)
MoCA	26.3 (2.55)	25.8 (2.54)	22.7 (3.25)	16.9 (4.60)
ADAS‐cog*	8.27 (4.23)	9.17 (4.45)	17.8 (6.78)	30.6 (8.19)
mPACC (DSST)	0.26 (2.67)	−0.16 (2.73)	−7.2 (4.04)	−15.4 (3.50)
mPACC (Trails B)	0.44 (2.51)	−0.25 (2.50)	−6.6 (3.80)	−14.4 (3.31)
LM immediate	14.2 (3.21)	15.0 (3.33)	8.1 (3.53)	4.0 (2.72)
LM delayed	13.3 (3.46)	13.0 (3.30)	5.4 (3.51)	1.4 (1.85)
RAVLT immediate	47.0 (10.3)	45.0 (9.94)	32.7 (9.79)	22.4 (7.31)
RAVLT 30 min	8.34 (4.12)	7.47 (3.90)	3.16 (3.42)	0.70 (1.65)
Trails A*	31.3 (11.1)	34.6 (11.1)	43.4 (21.3)	65.8 (37.2)
Trails B*	72.7 (35.5)	87.0 (41.9)	122.3 (67.6)	200.0 (87.1)
DSST	46.5 (9.58)	44.5 (8.83)	35.7 (10.8)	26.8 (12.8)
BNT	27.8 (2.63)	27.7 (2.16)	25.59 (3.88)	22.05 (6.18)
Animal fluency	21.5 (5.55)	20.8 (5.40)	17.0 (5.09)	12.3 (5.05)
Vegetables fluency	15.1 (3.66)	13.9 (3.37)	10.5 (3.42)	8.3 (3.43)
Clock drawing	4.73 (0.59)	4.63 (0.63)	4.25 (0.94)	3.26 (1.42)
Digit span f	9.05 (2.16)	8.52 (1.89)	8.28 (2.02)	7.54 (1.85)
Digit span b	7.45 (2.16)	6.80 (2.34)	6.12 (1.99)	4.74 (1.83)

Longitudinal follow‐up characteristics differed by clinical stage: median follow‐up duration was 4.0 years in both CN groups, 3.2 years in the Aβ+ MCI group, and 2.0 years in the Aβ+ dementia group, while the median number of study visits was 5 in both CN groups, 7 in the MCI group, and 4 in the dementia group.

Biomarker levels showed significant variations, with Aβ PET increasing from 0.0 in the Aβ‐ group to 92.4 in the Aβ+ dementia group. Tau PET similarly rose from 1.17 in Aβ‐ individuals to 1.71 in those with Aβ+ dementia. FAQ increased markedly, from 0.1 in Aβ‐ individuals to 13.1 in the dementia group. Cognitive performance also declined: MMSE scores dropped from 29.2 to 23.1, MoCA scores decreased from 26.3 to 16.9, and ADAS‐cog scores worsened from 8.27 to 30.6. The mPACC scores declined from approximately 0 in the Aβ‐ group to ‐15.4 and ‐14.4 in the Aβ+ dementia group.

Table 2 details the impact of demographic variables on cognitive test scores in CN Aβ‐ individuals. Higher education consistently correlated with better performance across most cognitive assessments. Increased age was associated with poorer performance on roughly half of the cognitive tests. Similarly, male sex was linked to lower scores in approximately half of the cognitive measures, indicating worse cognitive performance.

**TABLE 2:** Effect of demographic variables on cognitive measures in CN Aβ‐ individuals. NOTE. Numbers represent standardized β regression coefficients with outcome measures aligned so that higher values indicate worse performance. Abbreviations: Aβ‐, amyloid negative, ADAS‐cog, Alzheimer's Disease Assessment Scale; DSST, Digit symbol substitution test; mPACC, Preclinical Alzheimer Cognitive Composite; MMSE, Mini‐Mental State Examination; MoCA, Montreal Cognitive Assessment; RAVLT, Rey Auditory Verbal Learning Test.

Cognitive measure	Age	Male sex	Education
MMSE	0.02 (p = 0.628)	0.30 (p = 0.001)	−0.28 (p < 0.001)
MOCA	0.18 (p < 0.001)	0.36 (p = 40.002)	−0.22 (p < 0.001)
ADAS‐cog	0.10 (p = 0.028)	0.53 (p < 0.001)	−0.22 (p < 0.001)
mPACC (DSST)	−0.04 (p = 0.347)	0.50 (p < 0.001)	−0.43 (p < 0.001)
mPACC (Trails B)	0.02 (p = 0.692)	0.44 (p < 0.001)	−0.46 (p < 0.001)
LM immediate	−0.21 (p < 0.001)	0.44 (p < 0.001)	−0.36 (p < 0.001)
LM delayed	−0.23 (p < 0.001)	0.32 (p = 0.002)	−0.37 (p < 0.001)
RAVLT immediate	0.05 (p = 0.228)	0.56 (p < 0.001)	−0.24 (p < 0.001)
RAVLT 30 min	0.11 (p = 0.012)	0.38 (p < 0.001)	−0.18 (p = 0.002)
Trails A	0.23 (p < 0.001)	0.04 (p = 0.761)	−0.16 (p = 0.013)
Trails B	0.16 (p < 0.001)	0.04 (p = 0.753)	−0.29 (p < 0.001)
DSST	0.03 (p = 0.747)	0.19 (p = 0.414)	−0.46 (p < 0.001)
BNT	−0.07 (p = 0.176)	−0.26 (p = 0.166)	−0.23 (p = 0.016)
Animal fluency	0.18 (p < 0.001)	0.15 (p = 0.149)	−0.38 (p < 0.001)
Vegetables fluency	0.14 (p = 0.114)	0.76 (p < 0.001)	−0.16 (p = 0.122)
Clock drawing	0.11 (p = 0.014)	0.15 (p = 0.159)	−0.14 (p = 0.01)
Digit span b	0.08 (p = 0.398)	0.10 (p = 0.679)	−0.23 (p = 0.059)
Digit span f	0.14 (p = 0.158)	0.17 (p = 0.478)	−0.29 (p = 0.021)

### 3.2 Disease Progression Modeling

Figure 1 (top) presents the model results for staging individual patients based on their observed trajectories of Aβ and tau PET and functional impairment (FAQ). The model estimates indicated a time span of approximately 20 years from Aβ PET positivity to the latest stages of AD dementia. The distribution of diagnoses across the predicted timeline (Figure 1, bottom) suggested that Aβ+ CN is the most typical status from 2.1 years before to 6.2 years after predicted Aβ PET positivity, followed by Aβ+ MCI from 6.2 years to 13.6 years after predicted Aβ PET positivity, and finally, dementia after 13.6 years from predicted Aβ PET positivity.

### 3.3 Sensitivity of Cognitive Measures Across the AD Continuum

The observed longitudinal trajectories of composite scores demonstrated strong Spearman correlations with predicted disease time (Figure 2). Predicted disease time was highly correlated across measures. For the composite measures, Spearman correlations were as follows: MoCA (ρ = 0.69), MMSE (ρ = 0.73), ADAS‐cog (ρ = 0.79), mPACC (Trails) (ρ = 0.82), and mPACC (DSST) (ρ = 0.81). Together, these results underscore alignment across cognitive measures in relation to predicted disease progression. Observed longitudinal trajectories, with Spearman correlations ranging from ρ = 0.36–0.81, and corresponding quantile regression curves (25th, 50th, and 75th percentiles) for each cognitive measure across predicted disease time are provided (Figures S1–S22).

**FIGURE 2:**
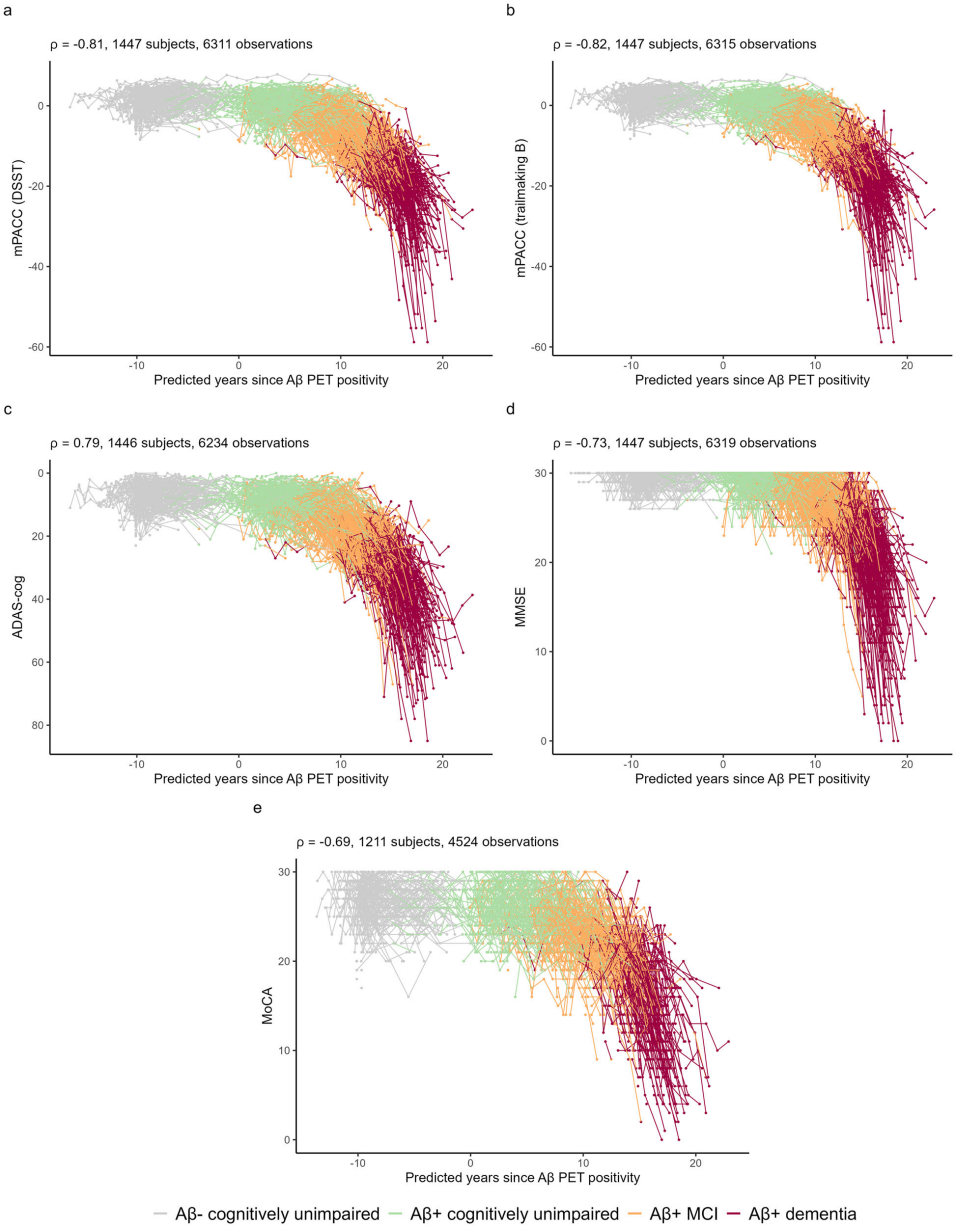
Observed longitudinal cognitive measure trajectories for screening tools and composite scores. ρ = Spearman correlation between cognitive measurements and the predicted disease time. Abbreviations: Aβ‐, amyloid negative; Aβ+, amyloid positive; ADAS‐cog, Alzheimer's Disease Assessment Scale; DSST, Digit symbol substitution test; MCI, Mild Cognitive Impairment; mPACC, Preclinical Alzheimer Cognitive Composite; MMSE, Mini‐Mental State Examination; MoCA, Montreal Cognitive Assessment; PET, positron emission tomography.

**FIGURE 3:**
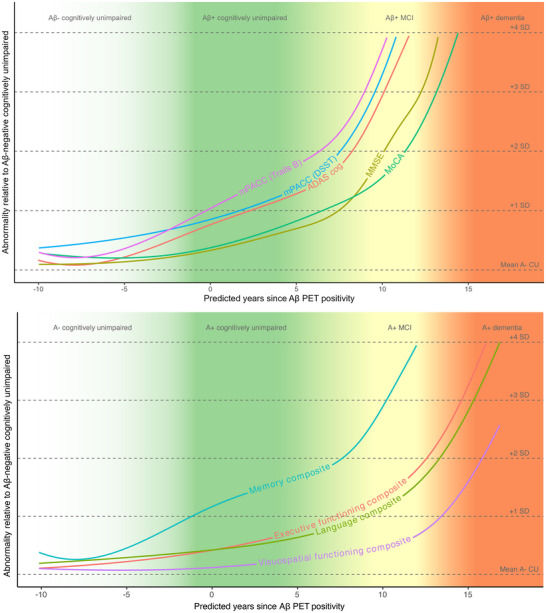
Trajectories showing abnormality of cognitive composite measures relative to amyloid‐negative cognitively normal individuals. The top panel shows cognitive composites. Bottom panel shows domain‐specific cognitive composites. Abnormality is defined by Aβ and tau PET imaging, and the severity of the Functional Activities Questionnaire. The white, green, yellow, and red bands represent clinical stages. Cognitive measures were adjusted for age, sex, and education prior to analysis and standardized against the mean (dashed lines) for Aβ‐ CN. Abbreviations: Aβ, amyloid; Aβ‐, amyloid negative; Aβ+, amyloid positive; ADAS‐cog, Alzheimer's Disease Assessment Scale; MCI, mild cognitive impairment; mPACC, Preclinical Alzheimer Cognitive Composite; MMSE, Mini‐Mental State Examination; MoCA, Montreal Cognitive Assessment; PET, positron emission tomography; SD, standard deviation.

**FIGURE 4:**
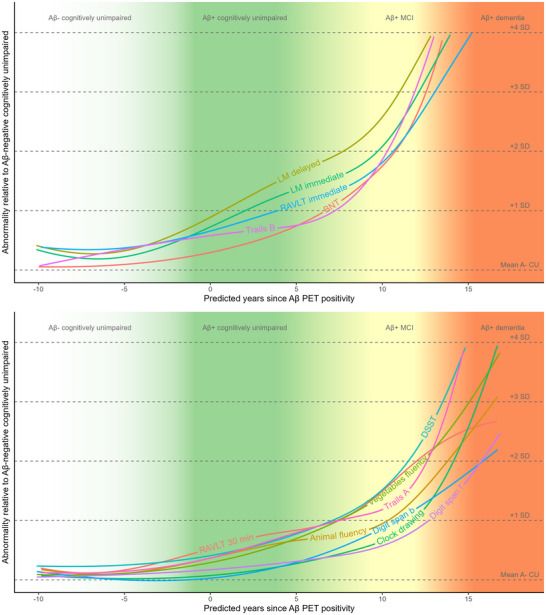
Trajectories showing abnormality of individual cognitive tests relative to amyloid‐negative cognitively normal individuals. The top panel shows individual cognitive tests with early abnormality. The bottom panel shows individual cognitive tests with later abnormality. Abnormality is defined by Aβ and tau PET imaging, and the severity of the Functional Activities Questionnaire. The white, green, yellow, and red bands represent clinical stages. Cognitive measures were adjusted for age, sex, and education prior to analysis and standardized against the mean (dashed lines) for Aβ‐ CN. Abbreviations: Aβ, amyloid; Aβ‐, amyloid negative; Aβ+, amyloid positive; DSST, Digit Symbol Substitution Test; LM, Logical Memory; MCI, mild cognitive impairment; PET, positron emission tomography; RAVLT, Rey Auditory Verbal Learning Test; SD, standard deviation; Trails, Trail Making Test.

To further characterize these relationships, Figures 3 and 4 present side‐by‐side trajectories of cognitive measures across the disease continuum. Figure 3 includes cognitive screening instruments, ADAS‐cog, and mPACC variants (top) alongside domain‐specific composites (bottom), while Figure 4 shows individual cognitive tests ordered by earlier (top) vs. later (bottom) abnormality. Across all measures, abnormalities increased progressively along the disease continuum, with the steepest decline observed during the MCI stage and the transition to dementia. Domain‐specific composites revealed a sequential pattern of decline: memory declined earliest, followed by executive function and language, whereas visuospatial function was primarily affected in the dementia stage. These results indicate that memory measures are most sensitive to early disease‐related changes, with other domains becoming progressively affected, reflecting a temporal sequence of cognitive decline.

**FIGURE 5:**
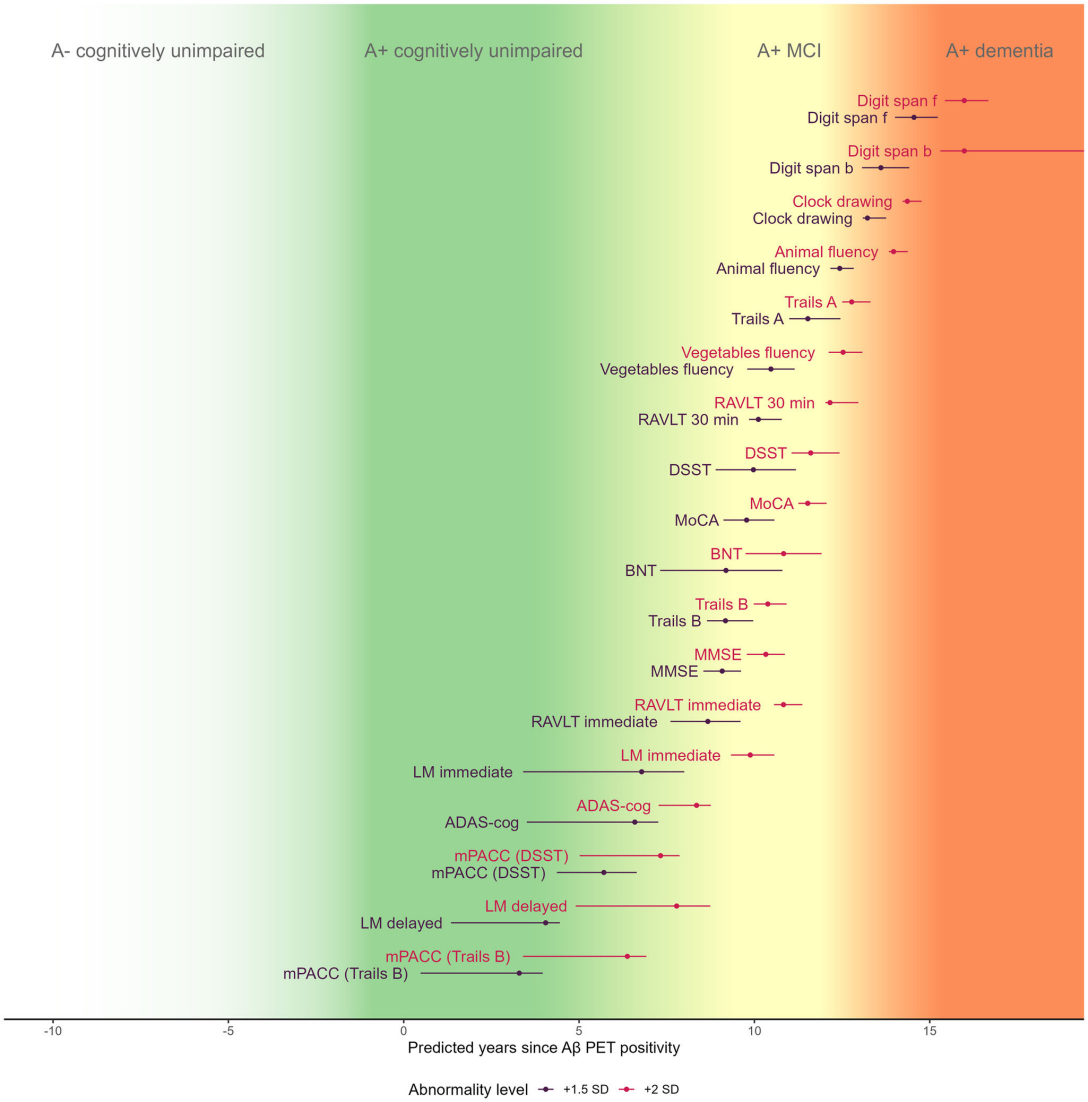
Estimated time point when cognitive measures on average reach different thresholds for abnormality relative to amyloid‐negative cognitively normal individuals. Abnormality is defined by Aβ and tau PET imaging, and the severity of the Functional Activities Questionnaire. The white, green, yellow, and red bands represent clinical stages. Cognitive measures were adjusted for age, sex, and education prior to analysis and standardized against the mean (dashed lines) for Aβ‐ CN. The lines are 95% confidence intervals as determined by an empirical case bootstrap method with 1.000 resamplings. Abbreviations: Aβ, amyloid; Aβ‐, amyloid negative; Aβ+, amyloid positive; ADAS‐cog, Alzheimer's Disease Assessment Scale; DSST, Digit symbol substitution test; LM, Logical Memory; MCI, Mild Cognitive Impairment; mPACC, Preclinical Alzheimer Cognitive Composite; MMSE, Mini‐Mental State Examination; MoCA, Montreal Cognitive Assessment; PET, positron emission tomography; RAVLT, Rey Auditory Verbal Learning Test; SD, standard deviation; Trails, Trail Making Test.

Using the continuous‐time disease progression model to stage individual patients, we estimated the mean trajectories of T‐scores across various cognitive measures. Figure 5 presents a comparative analysis of all cognitive measures across the disease timeline, highlighting the clinical stages and the specific points within each stage at which the tests become abnormal. To determine abnormality, individual tests were assessed in relation to the Aβ‐ CN group, employing two thresholds: 1.5 SD as the lower threshold and 2 SD as the higher threshold. As depicted in Figure 5 and the corresponding confidence intervals, the variability in the certainty of crossing these thresholds differs among tests, particularly at the lower abnormality threshold.

Among composite measures, both variants of mPACC exhibited the earliest abnormalities across the disease continuum. ADAS‐cog followed, with both measures showing lower‐threshold abnormalities during the CN stage and reaching the higher threshold in the MCI stage. In mPACC, lower‐threshold abnormalities emerged approximately 3–5 years post–Aβ positivity, with higher‐threshold abnormalities appearing 6–8 years post–Aβ positivity. By contrast, MoCA and MMSE first exhibited lower‐threshold abnormalities in the MCI stage (9–10 years post–Aβ positivity), with higher‐threshold abnormalities emerging near the transition from MCI to dementia (11–12 years post–Aβ positivity).

At the level of individual tests, LM Delayed was the first to exhibit lower‐threshold abnormalities during the CN phase, emerging approximately 4 years post–Aβ positivity, followed by LM Immediate and RAVLT immediate in the MCI phase, with all three reaching higher‐threshold abnormalities in the MCI stage. BNT was abnormal at the lower threshold in MCI, followed by Trails B, DSST, RAVLT 30, Vegetable fluency, and Trails A. These tests generally reached higher levels of abnormality during late MCI or at the MCI‐dementia transition. Animal fluency, Digit Span b, and Clock Drawing displayed lower levels of abnormality in late MCI, with higher levels emerging during the dementia stage. Finally, Digit Span f exhibited both lower and higher levels of abnormality in the dementia stage, approximately 16 years post–Aβ positivity.

## 4 Discussion

This study investigated the trajectory of decline across a wide range of cognitive measures in AD, spanning approximately 20 years from the preclinical stage to dementia. Using novel disease progression modeling, we incorporated data from Aβ and tau biomarkers alongside IADL measures to estimate a continuous trajectory of AD progression. Our analysis provided detailed information on the continuous progression of cognitive impairments in AD, including a comparison of when different cognitive tests become abnormal.

As AD progressed, both the severity and number of abnormal tests increased. As expected, episodic memory—the hallmark of early AD—was the first cognitive domain to show abnormality during disease progression, followed by abnormality in executive function, language, processing speed, and visuospatial abilities. This pattern aligns with previous studies [2, 3, 36] and underscores the importance of assessing memory in the early stages of AD. A broader array of cognitive tests should be employed in later stages to capture the full spectrum of cognitive decline.

The rate of cognitive decline accelerated along the disease continuum, with the most rapid abnormalities occurring at the MCI stage and during the transition from MCI to dementia, indicating a non‐linear trajectory. This pattern is consistent with previous research, which suggests a long preclinical phase before an increasing rate of impairment observed in the years preceding the dementia stage [37, 38].

Importantly, our continuous trajectory model over the course of the disease allows us to illustrate not only which tests and domains are affected first but also more precisely when these changes occur within clinical stages. Our findings indicate that the typical stages of AD often span several years and that there is considerable variability in cognitive decline within each stage. For example, Trails B became abnormal during the early MCI stage, whereas Trails A reached the same level of abnormality in the late MCI stage, approximately 3 years later. While the clinical staging of AD offers a useful structure for comprehending the progression of cognitive decline [39], it is important to recognize the potential oversimplification inherent in these stages.

The cognitive composite PACC became abnormal first in the disease timeline, emphasizing the relevance of cognitive composites in cognitive assessment early in the disease process. Multi‐domain cognitive composite measures, like the PACC [23], have been developed to enhance the assessment of cognitive changes in preclinical AD stages, and have been validated [40, 41] and optimized [42] in numerous studies. While the underlying reasons for this pattern are likely multifactorial and discussed elsewhere, measurement characteristics—such as reliability—may contribute, as composite scores can reduce random variability and improve signal detection by averaging across tests. Our findings further indicate that the two variants of PACC may have slightly differing sensitivities in the early AD stages, consistent with previous findings [43].

The domain‐specific composites revealed a staggered progression of cognitive decline. Memory deficits emerged first in the CN stage and progressed in early MCI, followed by executive dysfunction in MCI that intensified in late MCI. Language impairments appeared in late MCI and became more pronounced in dementia, while visuospatial deficits were mainly observed in the dementia stage. These patterns underscore the sequential emergence of cognitive impairments across different domains throughout the AD continuum.

While the overall pattern aligns with expectations, some individual cognitive tests displayed unexpected sensitivity, particularly in RAVLT and verbal fluency tests. RAVLT delayed recall, typically considered an early episodic memory measure, became abnormal later than LM, which showed earlier deviations along the disease timeline. Similarly, verbal fluency tests (Animal and Vegetables fluency) did not show abnormalities during the preclinical stages, despite prior evidence suggesting that semantic memory decline can be detected early in AD [42, 44].

Abnormalities in visuospatial function tests were predominantly observed in the later stages of AD, particularly in the dementia stage. This may reflect the limited availability of tests in ADNI specifically designed to assess visuospatial functions. However, other studies have explored other aspects of visuospatial function, including non‐constructional visuospatial test performance like spatial navigation [45]. Furthermore, research suggests that tests like the Digital Clock Drawing Test, when enhanced by artificial intelligence, may provide valuable information [46].

Beyond test‐specific sensitivities, participant characteristics such as age, sex, and education also influenced performance, underscoring the need for demographically adjusted norms, particularly for single‐assessment testing. However, it is important to note that demographic adjustments can be problematic if outdated norms are used [47]. Moreover, demographic adjustments in cognitive testing have faced criticism for potentially reducing discriminatory power, as factors like age—frequently adjusted for—are also risk factors for cognitive impairment [48]. To mitigate these concerns, we normalized scores using data from Aβ‐, CN individuals, thereby reducing the risk of diminishing the performance of the tests.

In our disease timeline, individuals’ Aβ biomarker statuses (Figure 1) are not fully concordant with predicted time of PET Aβ+ (e.g., some are positive before predicted time of PET Aβ+, and some remain negative a while after predicted time of PET Aβ+). This is to be expected due to two different factors. The first reason is that Aβ status is both determined by PET and CSF (Aβ42/40 ratio), and it has been suggested that the CSF+/PET− status may arise because CSF can detect Aβ at an earlier stage [49, 50]. This is supported by higher rates of CSF+/PET− cases compared to CSF−/PET+ cases across several studies [51, 52, 53]. In line with this, in our data, we observed a higher proportion of CSF positivity compared with PET positivity at earlier estimated disease stages. The second reason is that the model predicts the most likely time since PET Aβ+ for each subject based on all their data, and thus weights the signal‐to‐noise level in Aβ PET biomarkers against the signal‐to‐noise in tau PET and FAQ to find the best overall fit, sometimes sacrificing fidelity in Aβ PET.

Importantly, the proposed disease timeline demonstrates sensitivity across the entire AD continuum, beginning with the earliest detectable amyloid accumulation and remaining independent of any single cognitive test. In this timeline, Aβ PET is sensitive in the preclinical phases, tau PET is sensitive in the MCI and mild dementia phase, and the FAQ is sensitive in the dementia phase. A key strength of our approach is the integration of the FAQ as a functional outcome measure alongside Aβ and tau PET, ensuring that disease progression is captured through both biomarker changes and functional impairment.

To illustrate the full range of model behavior, figures include individuals across the entire estimated disease timeline, including those with predicted negative disease times. These individuals show no evidence of amyloid accumulation and are therefore effectively placed at the extreme preclinical end of the timeline. While predicted times in this range are not meaningful as indicators of disease stage, their inclusion demonstrates the model's ability to accommodate variability in sub‐threshold biomarker and cognitive measures. Accordingly, interpretation of disease stage and timing should be restricted to participants with predicted positive disease times, where placement reflects meaningful progression along the AD continuum.

While our study has strengths, it also has limitations. First, the data is sourced exclusively from the ADNI database, which is limited to North America and Canada, potentially restricting the generalizability of our findings [54].

Second, we did not explore potential phenotypic variants within the AD spectrum. Research suggests distinct trajectories of tau deposition [55] and atrophy patterns [56] resulting in varied cognitive impacts. Early‐onset AD often presents with language, visuospatial, and dysexecutive manifestations, whereas late‐onset AD typically presents with an amnestic syndrome [57]. Furthermore, our study does not encompass co‐pathologies. For instance, Lewy Body co‐pathology is associated with distinct cognitive profiles and clinical trajectories [58].

Third, longitudinal studies have demonstrated significant variability in cognitive decline rates among individuals with AD [59, 60]. Our analysis, however, does not account for this variability, as accurately modeling these variations across numerous outcomes remains challenging and represents a notable limitation of current approaches. Additionally, studies may overestimate the impact of differences in decline rates, as much of the observed variability may be attributable to individuals' positions within different stages of the disease, rather than true differences in the rate of decline. Nevertheless, the assumption in our disease progression model—that all patients progress at the same pace—is an oversimplification.

Fourth, an important consideration is our exclusion of participants with suspected non‐AD cognitive impairment, defined by a diagnosis of MCI or dementia, a CDR global score above 0, or an MMSE score below 26 with concurrent negative Aβ biomarker results. While this exclusion effectively removes non‐AD cases, it may have led to overestimated cognitive performance for the reference population, defined here as Aβ‐ CN.

Fifth, our study does not cover recent advancements in cognitive assessment, such as various digital methods [61, 62]. These include, but are not limited to, remote cognitive assessments administered via tablets and smartphones, passive monitoring through sensors and wearables, and spoken language analysis with automated language processing.

To conclude, our study highlights the varying sensitivity of cognitive tests throughout AD progression, with implications for clinical practice and trial design. Healthcare professionals can use these findings to select appropriate tests for each disease stage and interpret test scores to determine the likely clinical stage of a patient. Additionally, selecting appropriate cognitive endpoints and inclusion criteria in clinical trials is essential for accurately evaluating treatment efficacy at different stages of the disease. We emphasize the importance of personalized approaches and the consideration of demographic factors in clinical practice.

Future research should leverage more diverse cohorts, such as ADNI4 [63], assess how phenotypic variation and comorbidities—including vascular or other coexisting conditions—affect AD progression, and evaluate novel digital cognitive measures [64] (e.g., remote assessments, wearable sensors, automated speech analysis) as scalable tools for assessing sensitivity along the disease continuum and detecting subtle early changes.

## Funding

MS receives funding from the Knut and Alice Wallenberg Foundation (Wallenberg Centre for Molecular and Translational Medicine; KAW2014.0363 and KAW2023.0371), the Swedish Research Council (2017‐02869, 2021–02678, 2021–06545 and 2023–06188), the European Union's Horizon Europe research and innovation program under grant agreement no 101132933 (AD‐RIDDLE) and 101112145 (PROMINENT), the National Institute of Health (R01 AG081394‐01), Gates Ventures, the National Research Foundation of Korea (RS‐2023‐00263612), the Swedish state under the agreement between the Swedish government and the County Councils, the ALF‐agreement (ALFGBG‐813971 and ALFGBG‐965326), the Swedish Brain Foundation (FO2021‐0311 and FO2024‐0372), the Swedish Alzheimer Foundation (AF‐994900), the Sahlgrenska Academy at the University of Gothenburg, the Västra Götaland Region R&D (VGFOUREG‐995510) and Innovation platforms, Sahlgrenska Science Park and the National Institute for Health and Care Research, and University College London Hospitals Biomedical Research Centre.

## Consent Statement

The ADNI study received approval from the institutional review boards at each participating center, and all participants provided written informed consent.

## Conflicts of Interest

FÖ declares no conflict of interest. LLR is an employee and shareholder of Eli Lilly and Company. MS has served on advisory boards for Novo Nordisk, Lilly and Roche, received speaker honoraria from Bioarctic, Eisai, Genentech, Novo Nordisk, Lilly, Roche and Triolabs and receives research support (to the institution) from Beckman‐Coulter, Bioarctic, Novo Nordisk and Roche. He is a co‐founder of Centile Bioscience and serves as Associate Editor with Alzheimer’s Research & Therapy.

## Supporting information




**Supporting File**: advs74998‐sup‐0001‐SuppMat.docx.

## Data Availability

Data used in this study were obtained from the ADNI database (https://adni.loni.usc.edu). ADNI data are publicly available to qualified researchers upon registration and approval through the ADNI Data Use Agreement.
