# Supplementary material for: Cognitive Trajectories from Preclinical Alzheimer's Disease to Dementia
Source: Adv Sci (Weinh). 2026 Mar 30;13(32):e18124. doi: 10.1002/advs.202518124 (PMC13252657; doi:10.1002/advs.202518124)
Supplement: Supplementary file 1 — Supporting File: advs74998‐sup‐0001‐SuppMat.docx. [file ADVS-13-e18124-s001.docx]

Supporting Information

**Cognitive Trajectories from Preclinical Alzheimer’s Disease to Dementia**

Fredrik Öhman^a,b,c ‡^, Lars Lau Raket^d, ‡^, Michael Schöll^a,b,c *,^, for the Alzheimer’s Disease Neuroimaging Initiative†

^a^Department of Psychiatry and Neurochemistry, Institute of Neuroscience and Physiology, Sahlgrenska Academy, University of Gothenburg, Wallinsgatan 6, 431 41, Mölndal, Sweden
^b^Wallenberg Centre for Molecular and Translational Medicine, University of Gothenburg, Erik Dahlbergsgatan 11B, 411 26, Gothenburg, Sweden

^c^Department of Neuropsychiatry, Sahlgrenska University Hospital, Region Västra Götaland, Wallinsgatan 6, 431 41, Mölndal, Sweden

^d^Clinical Memory Research Unit, Lund University, S:t Johannesgatan 8, 211 46, Malmö, Sweden

*Corresponding author: Michael Schöll. E-mail address: [michael.scholl@gu.se](mailto:michael.scholl@gu.se)
Wallenberg Centre for Molecular and Translational Medicine and the Department of Psychiatry and Neurochemistry, University of Gothenburg, Wallinsgatan 6, 431 41 Mölndal, Sweden.

^‡^contributed equally

This PDF file includes: S1-S22


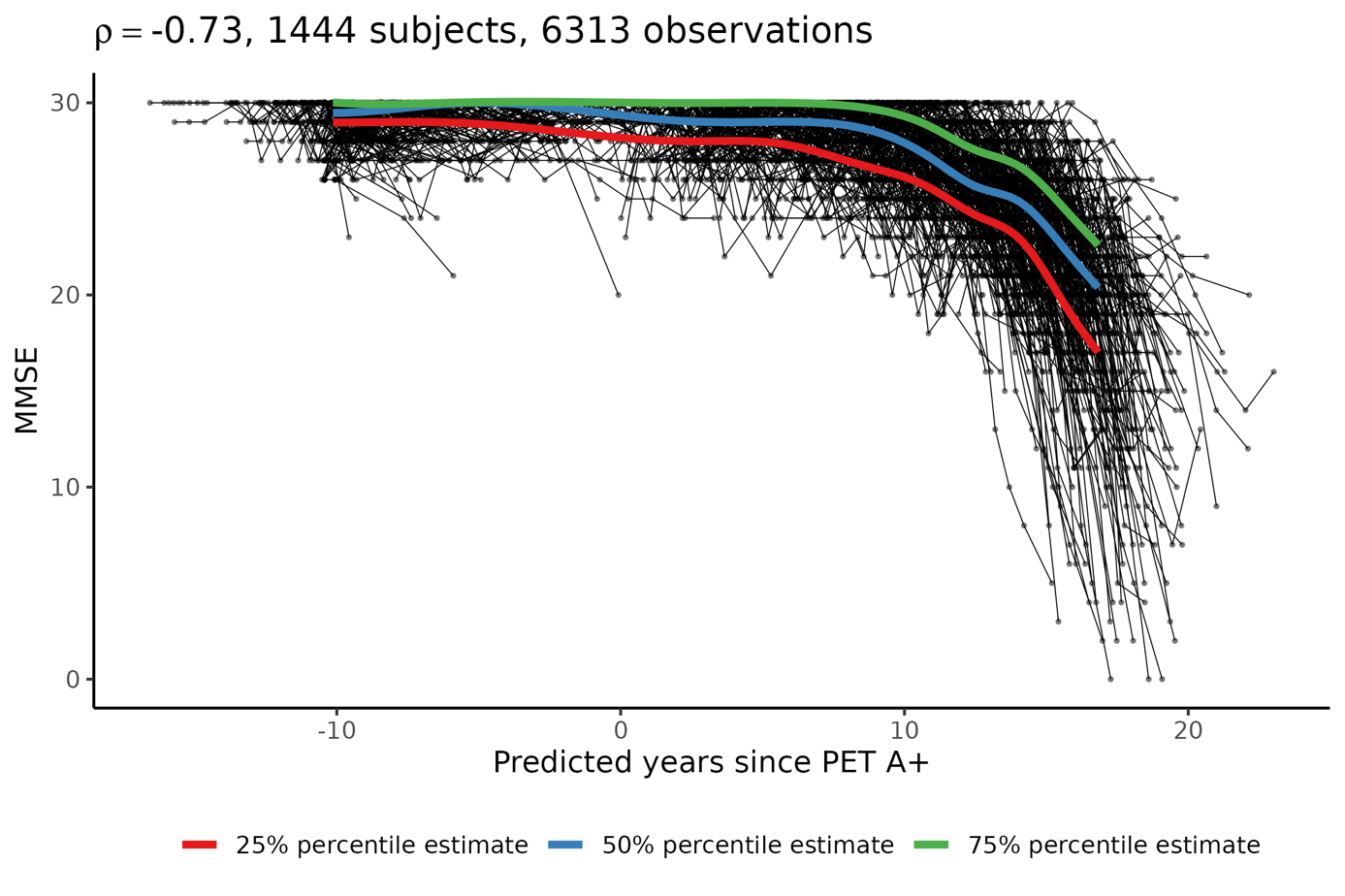


**Figure S1.** **Trajectories of Mini-Mental State Examination (MMSE) across predicted disease time.** Observed longitudinal trajectories and quantile regression curves (25th, 50th, 75th percentiles) plotted against predicted years since amyloid PET positivity.


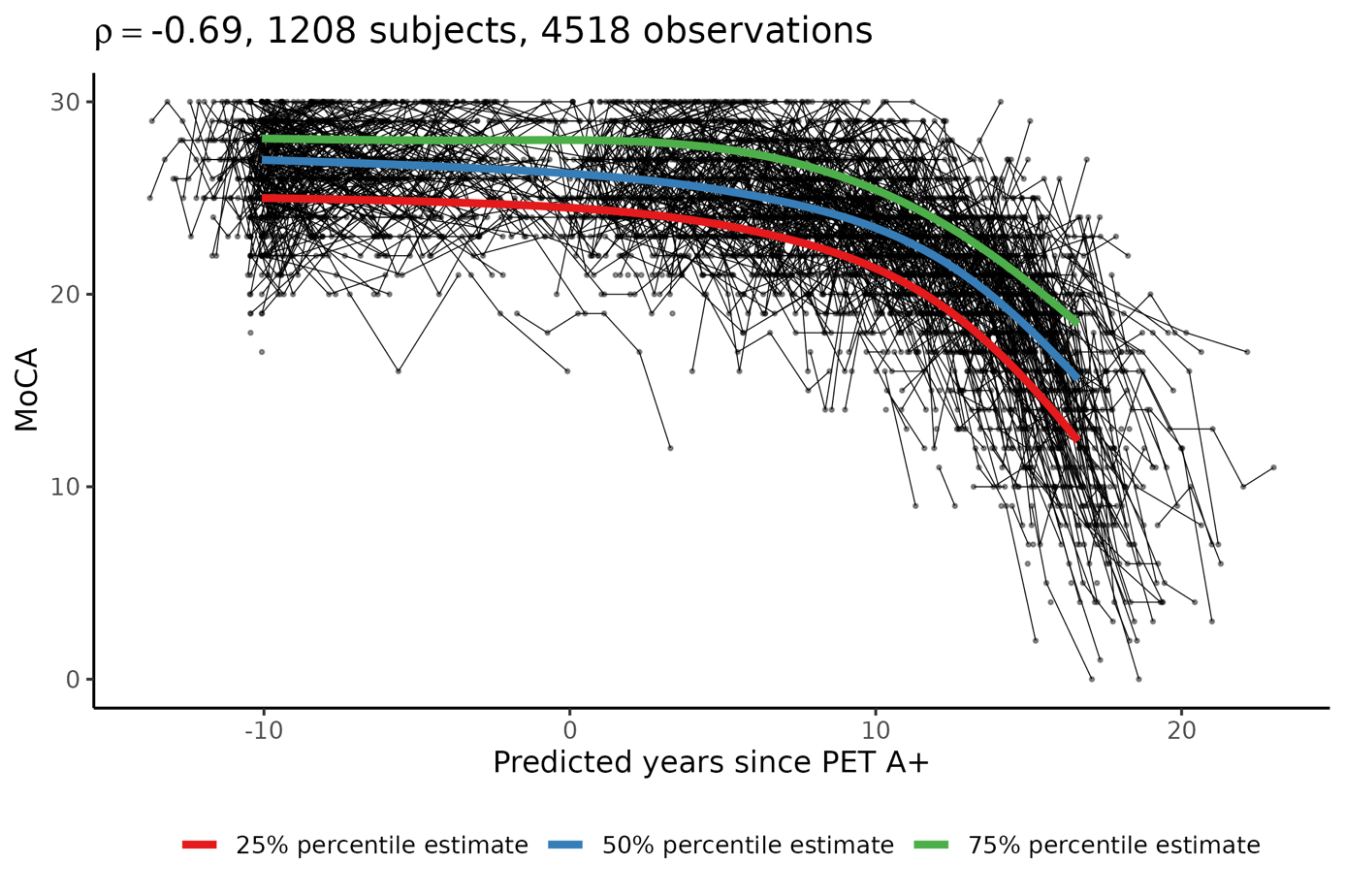


**Figure S2.** **Trajectories of Montreal Cognitive Assessment (MoCA) across predicted disease time.** Observed longitudinal trajectories and quantile regression curves (25th, 50th, 75th percentiles), plotted against predicted years since amyloid PET positivity.


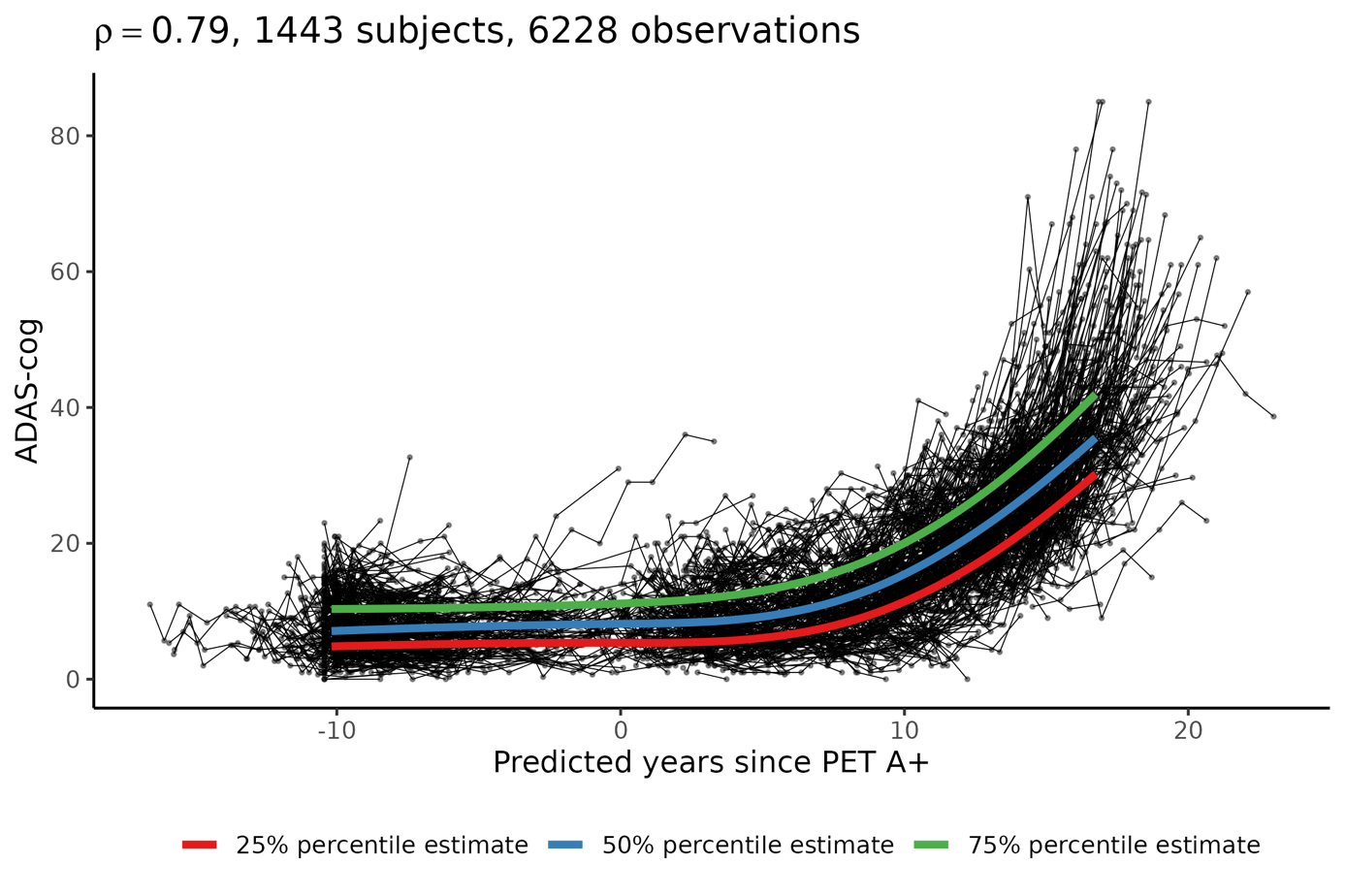


**Figure S3.** **Trajectories of Alzheimer’s Disease Assessment Scale–Cognitive Subscale (ADAS-cog) across predicted disease time.** Observed longitudinal trajectories and quantile regression curves (25th, 50th, 75th percentiles) plotted against predicted years since amyloid PET positivity.


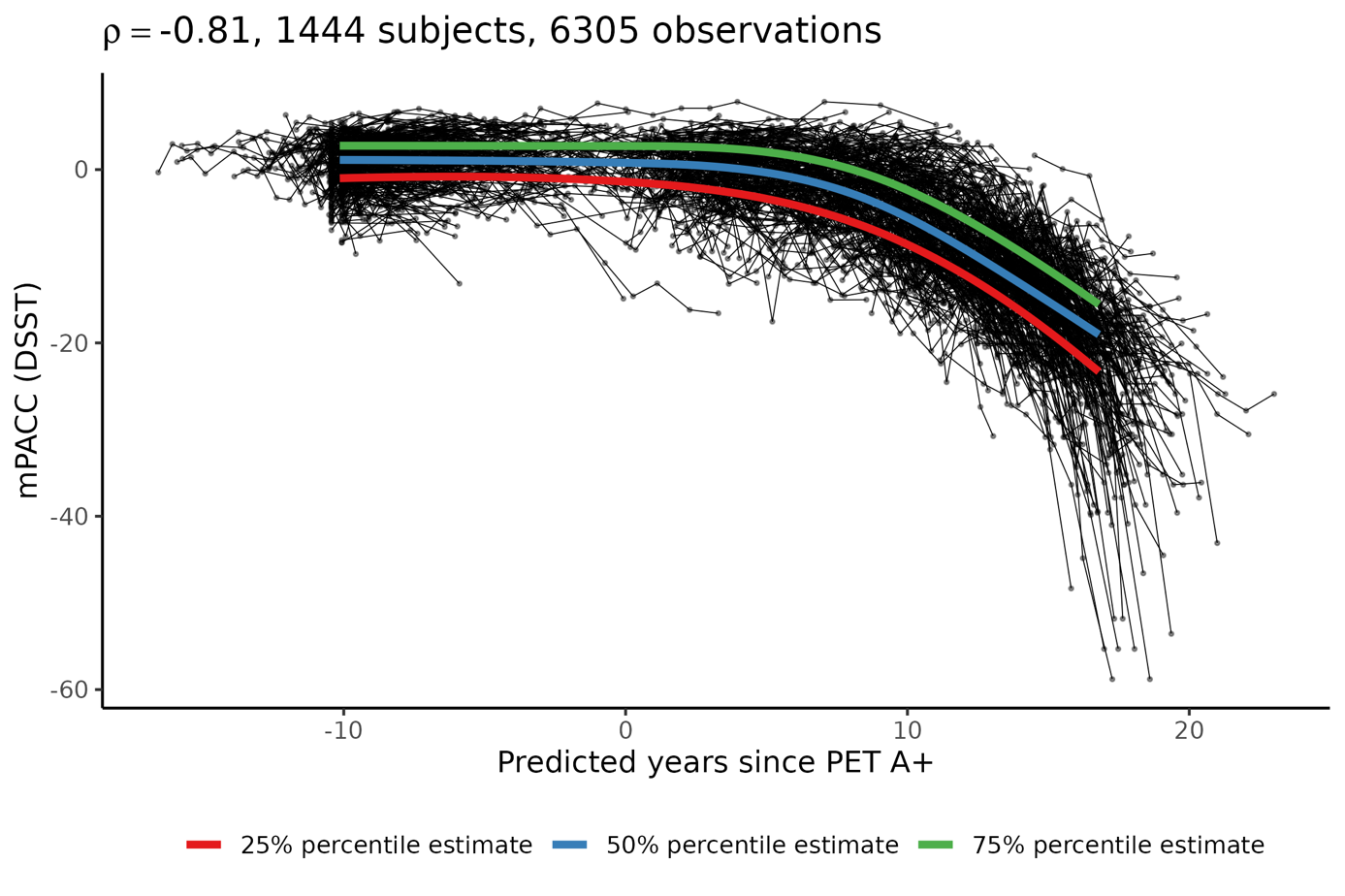


**Figure S4.** **Trajectories of modified Preclinical Alzheimer Cognitive Composite (mPACC, DSST variant) across predicted disease time.** Observed longitudinal trajectories and quantile regression curves (25th, 50th, 75th percentiles) plotted against predicted years since amyloid PET positivity.


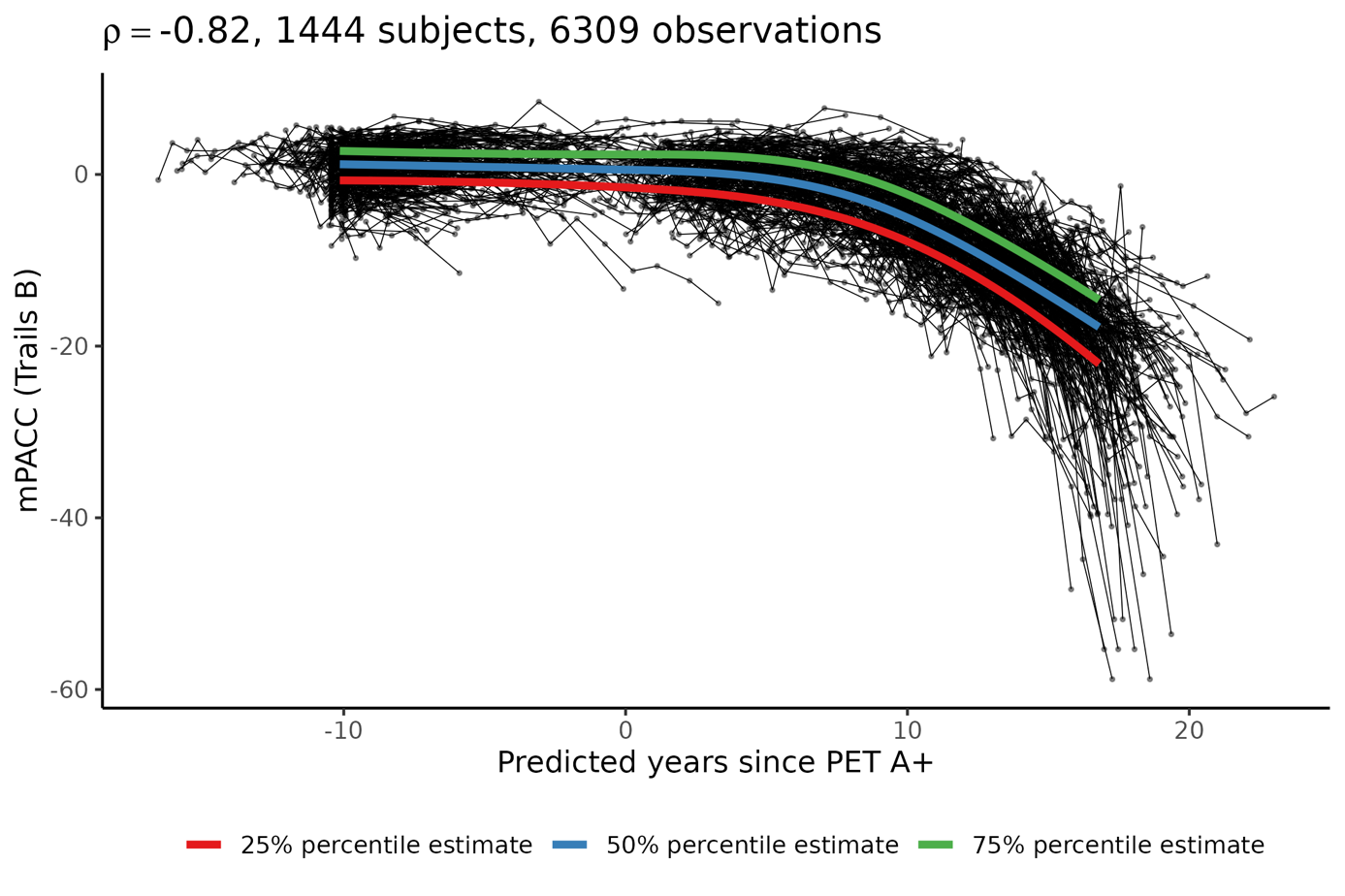


**Figure S5.** **Trajectories of modified Preclinical Alzheimer Cognitive Composite (mPACC, Trails B variant) across predicted disease time.** Observed longitudinal trajectories and quantile regression curves (25th, 50th, 75th percentiles) plotted against predicted years since amyloid PET positivity.


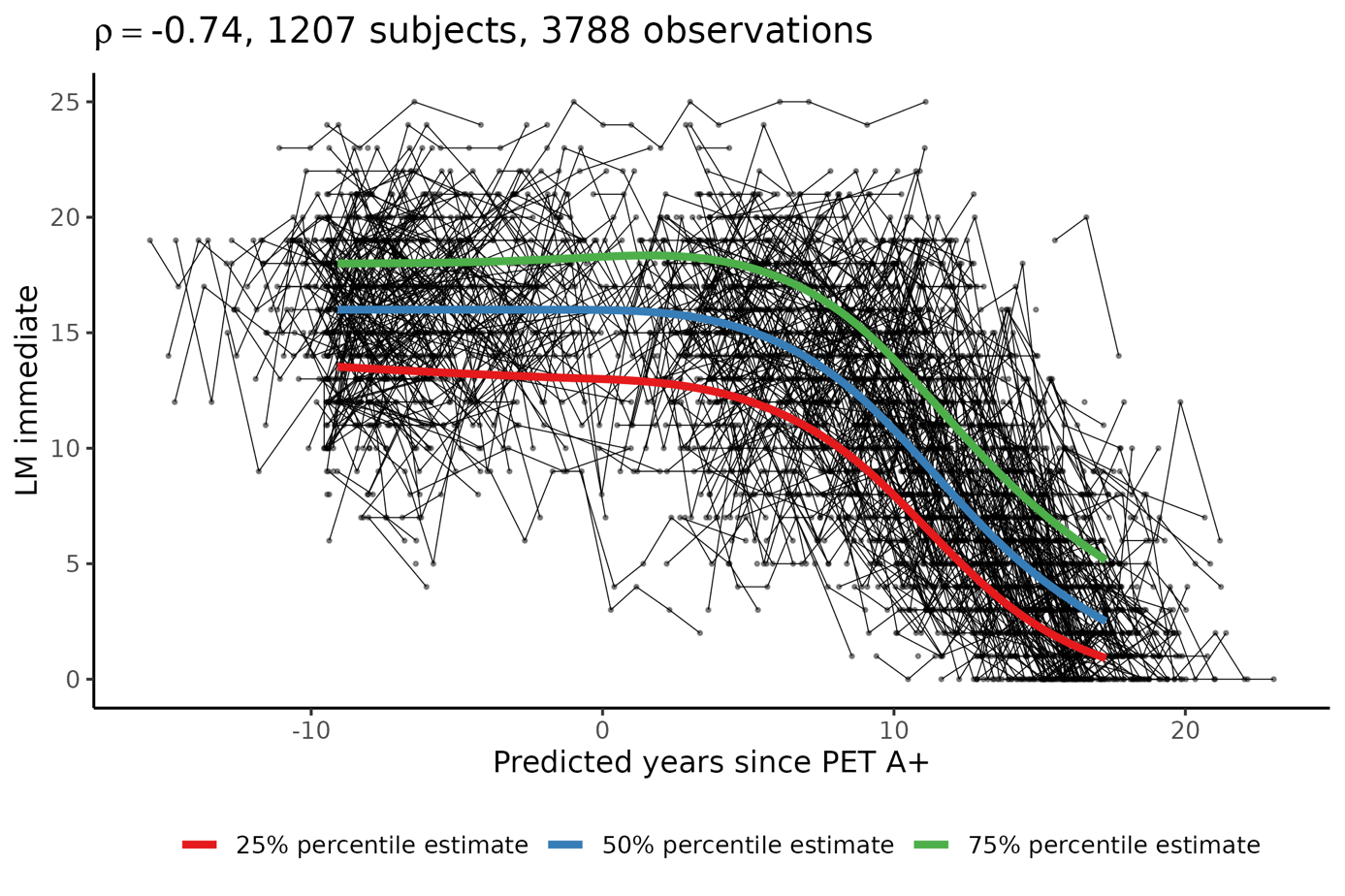


**Figure S6.** **Trajectories of Logical Memory Immediate Recall (LM immediate) across predicted disease time.** Observed longitudinal trajectories and quantile regression curves (25th, 50th, 75th percentiles) plotted against predicted years since amyloid PET positivity.


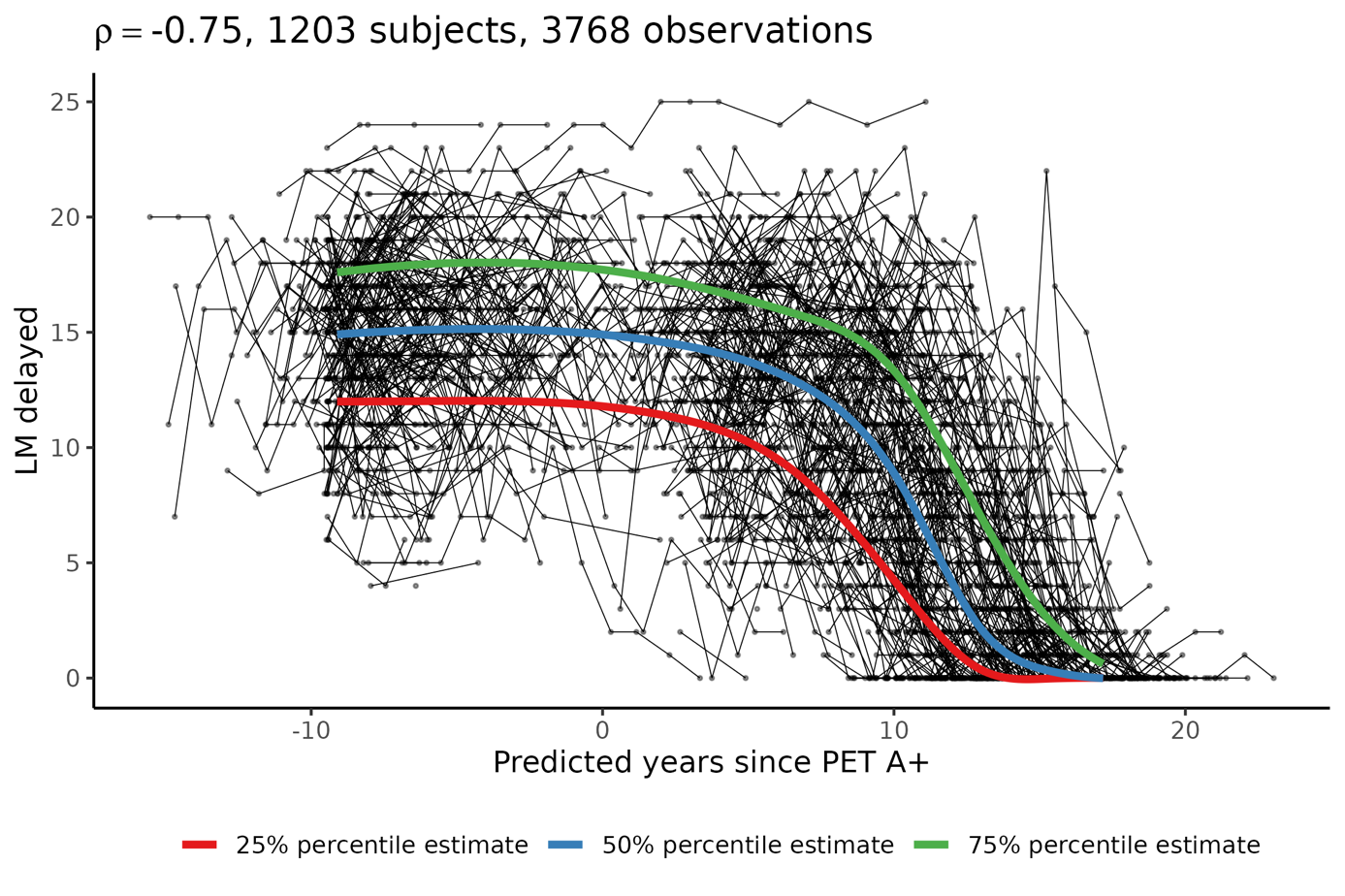


**Figure S7.** **Trajectories of Logical Memory Delayed Recall (LM delayed) across predicted disease time.** Observed longitudinal trajectories and quantile regression curves (25th, 50th, 75th percentiles) plotted against predicted years since amyloid PET positivity.

**
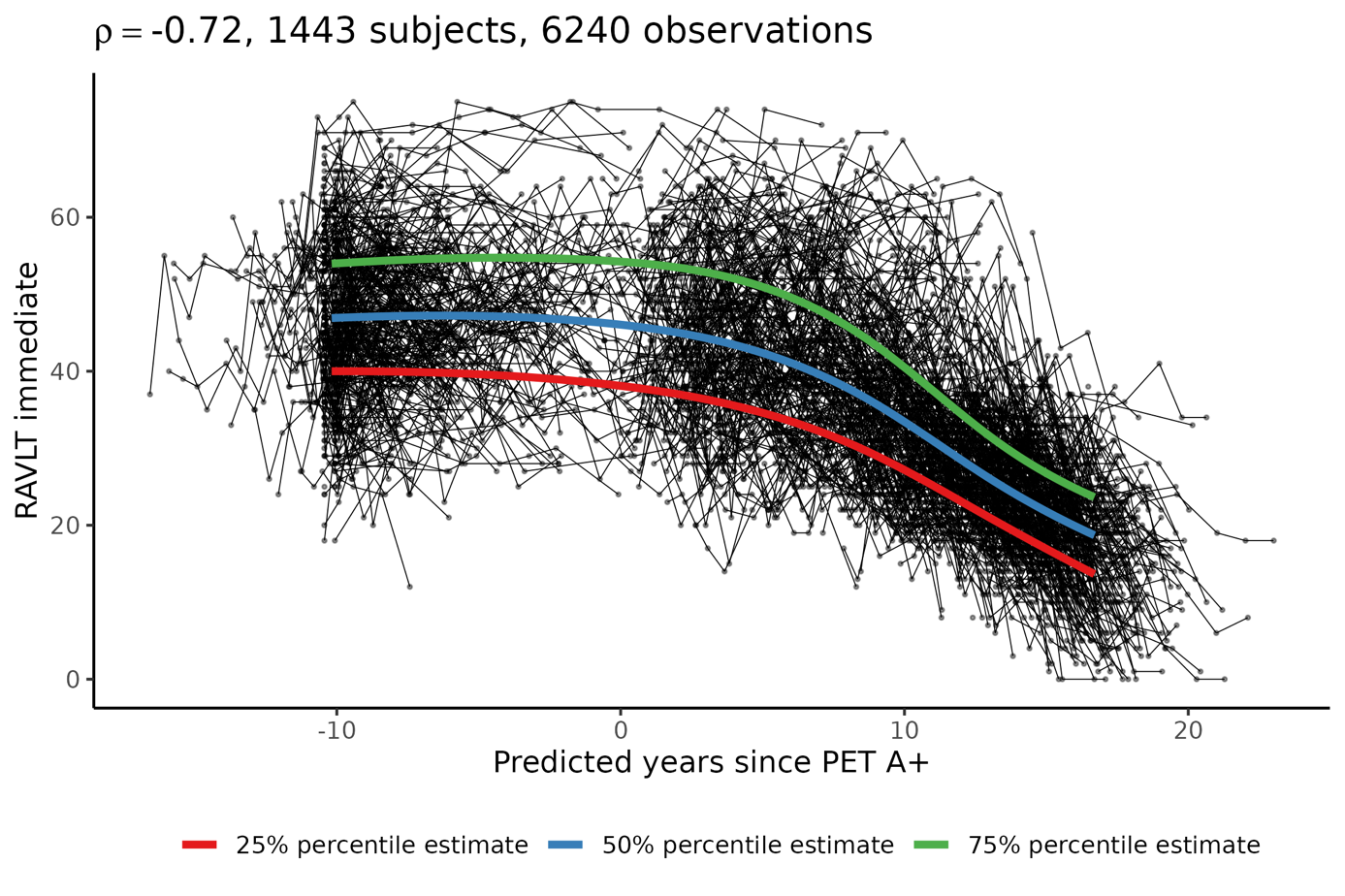
**

**Figure S8.** **Trajectories of Rey Auditory Verbal Learning Test, Trials 1–5 (RAVLT immediate) across predicted disease time.** Observed longitudinal trajectories and quantile regression curves (25th, 50th, 75th percentiles) plotted against predicted years since amyloid PET positivity.


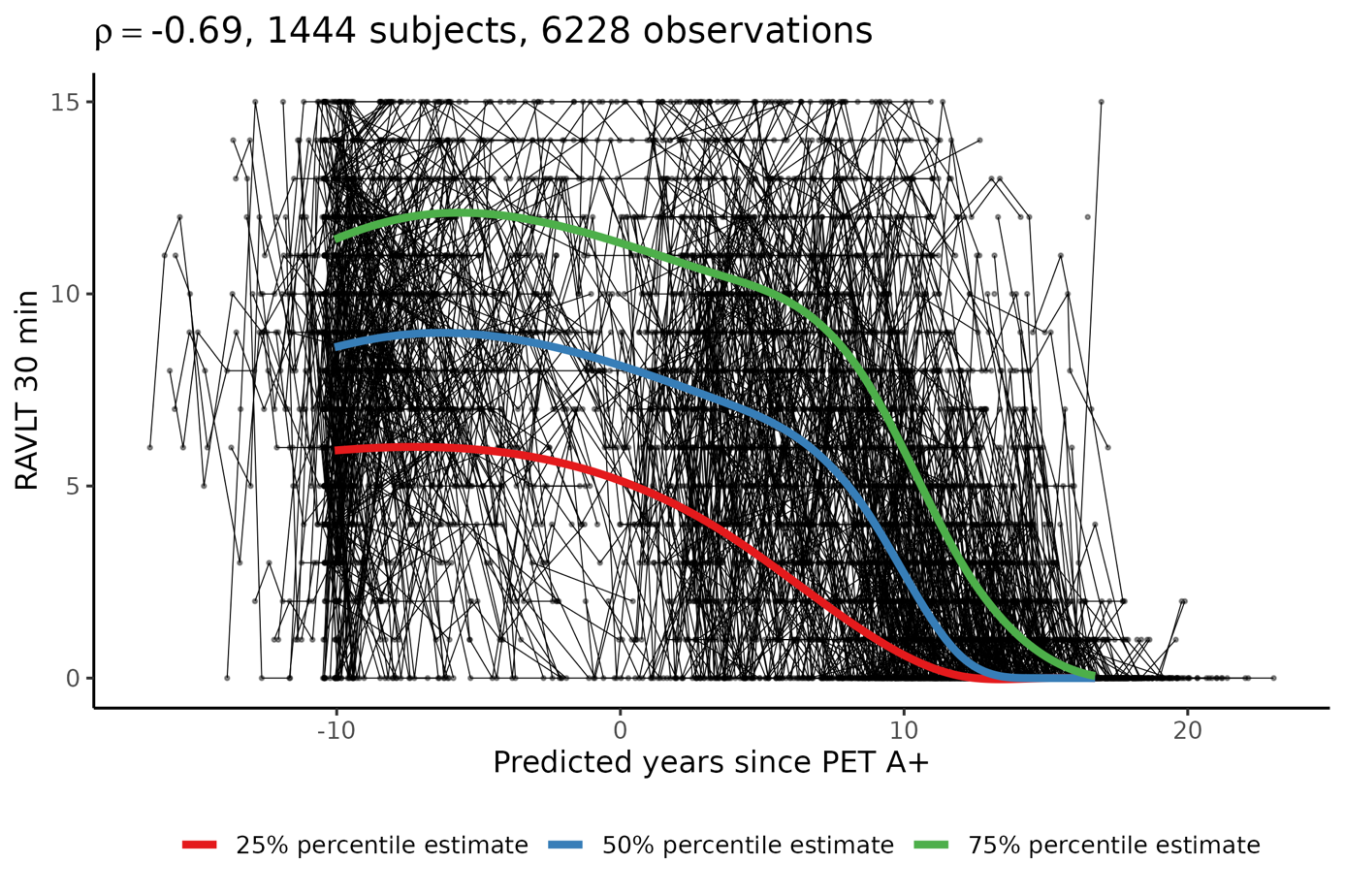


**Figure S9.** **Trajectories of Rey Auditory Verbal Learning Test, 30-minute Delayed Recall (RAVLT 30 min) across predicted disease time.** Observed longitudinal trajectories and quantile regression curves (25th, 50th, 75th percentiles) plotted against predicted years since amyloid PET positivity.


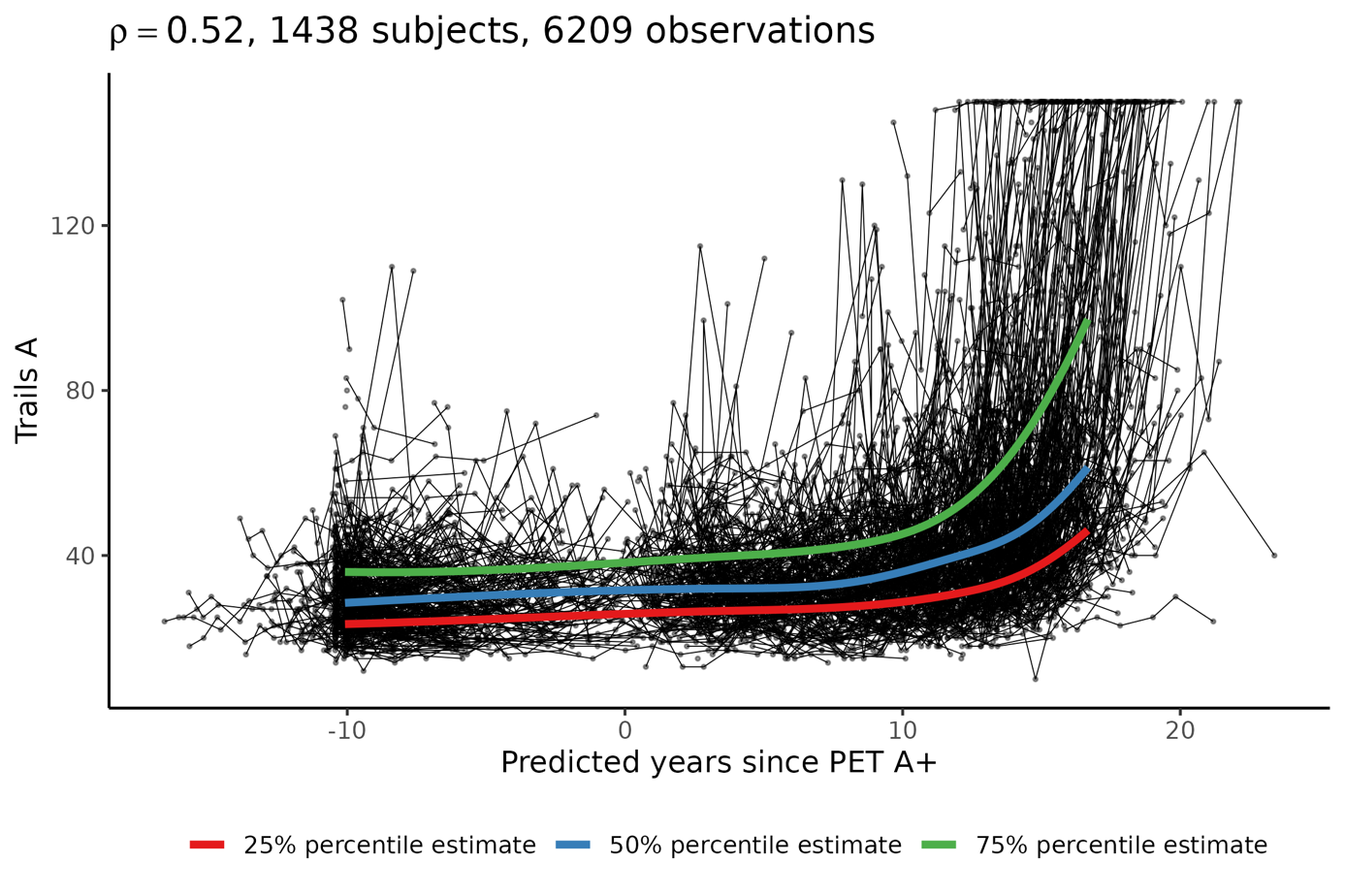


**Figure S10.** **Trajectories of Trail Making Test, Part A (Trails A) across predicted disease time.** Observed longitudinal trajectories and quantile regression curves (25th, 50th, 75th percentiles) plotted against predicted years since amyloid PET positivity.


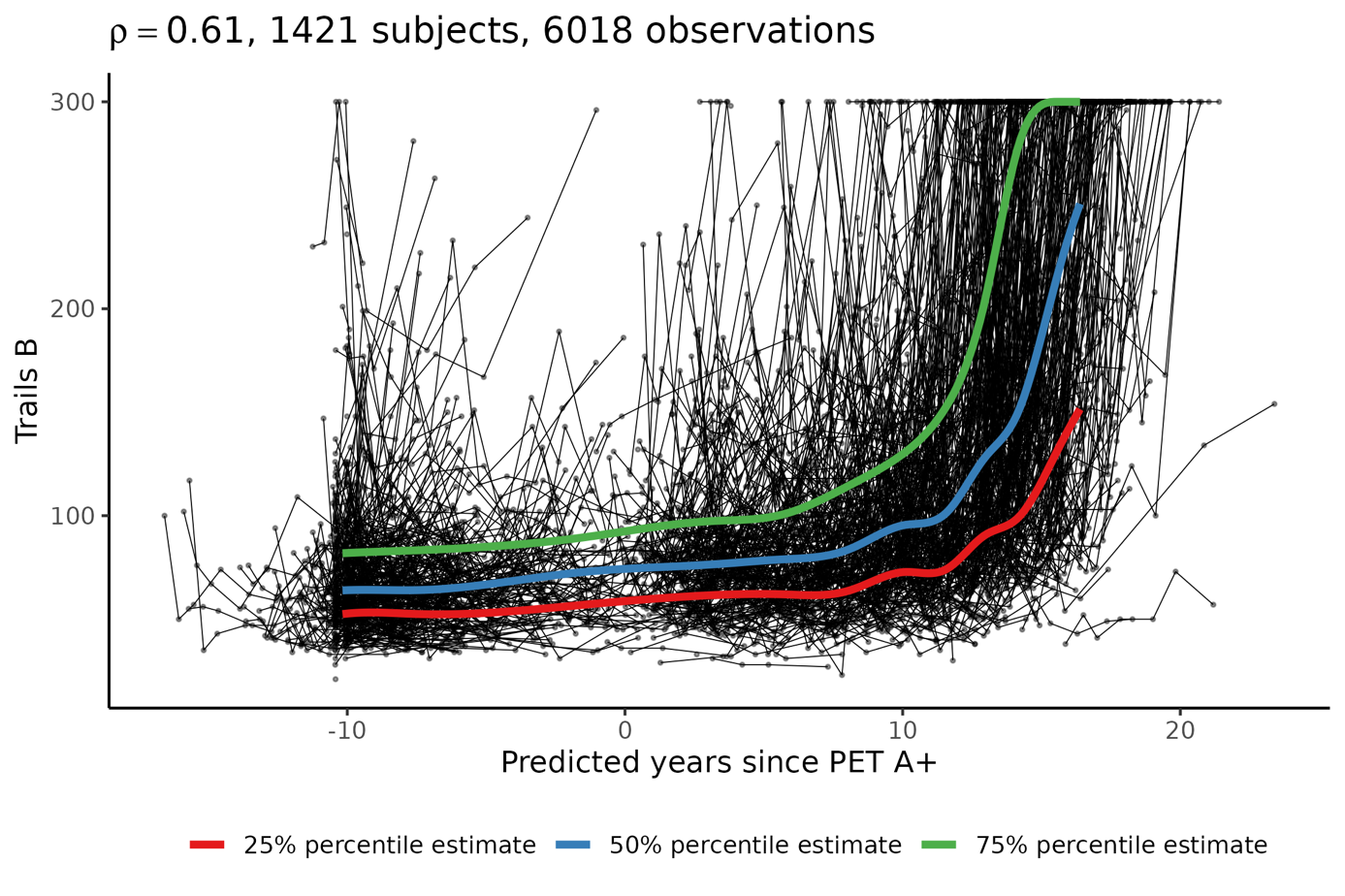


**Figure S11.** **Trajectories of Trail Making Test, Part B (Trails B) across predicted disease time.** Observed longitudinal trajectories and quantile regression curves (25th, 50th, 75th percentiles) plotted against predicted years since amyloid PET positivity.

**
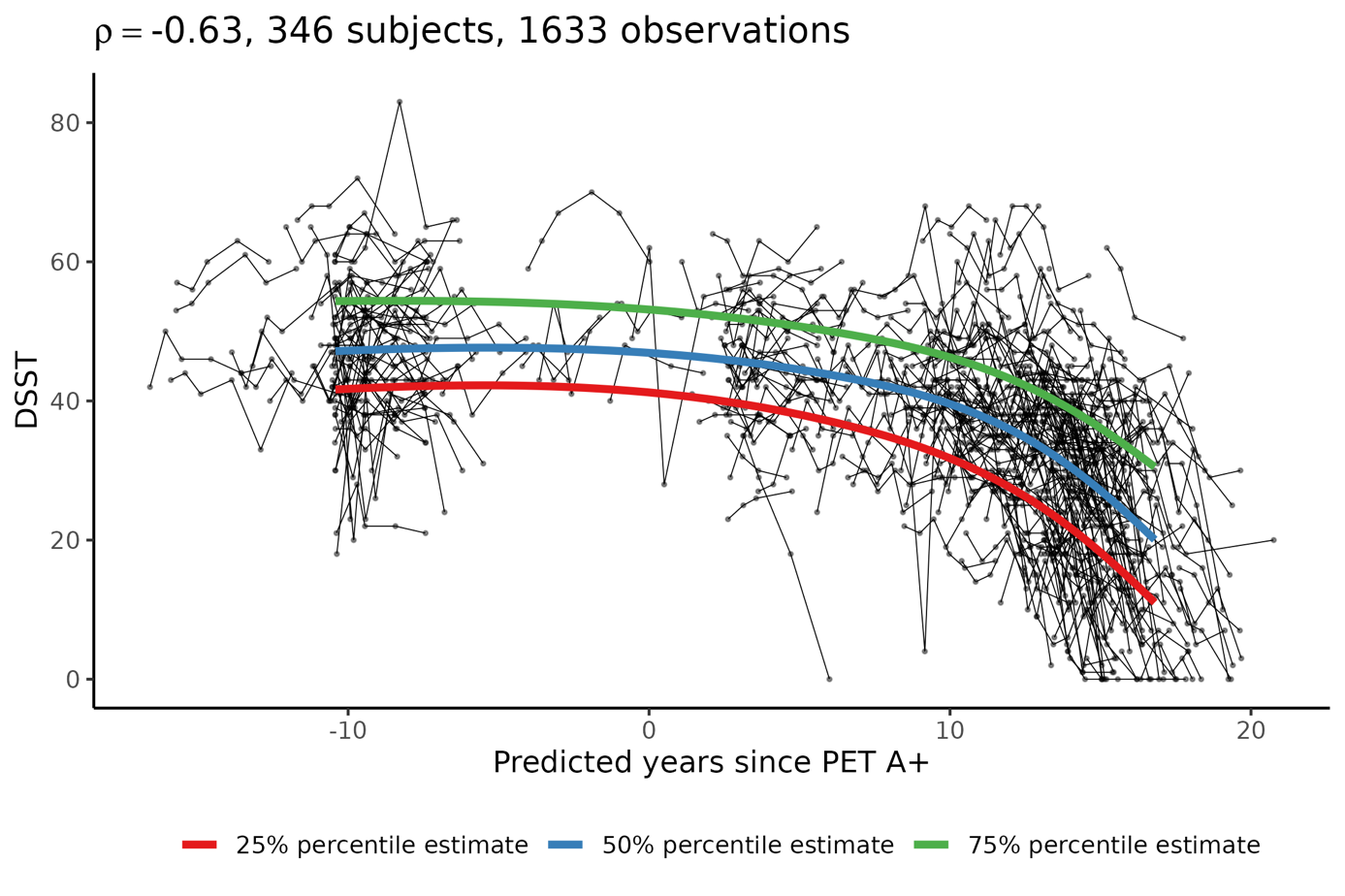
**

**Figure S12.** **Trajectories of Digit Symbol Substitution Test (DSST) across predicted disease time.** Observed longitudinal trajectories and quantile regression curves (25th, 50th, 75th percentiles) plotted against predicted years since amyloid PET positivity.


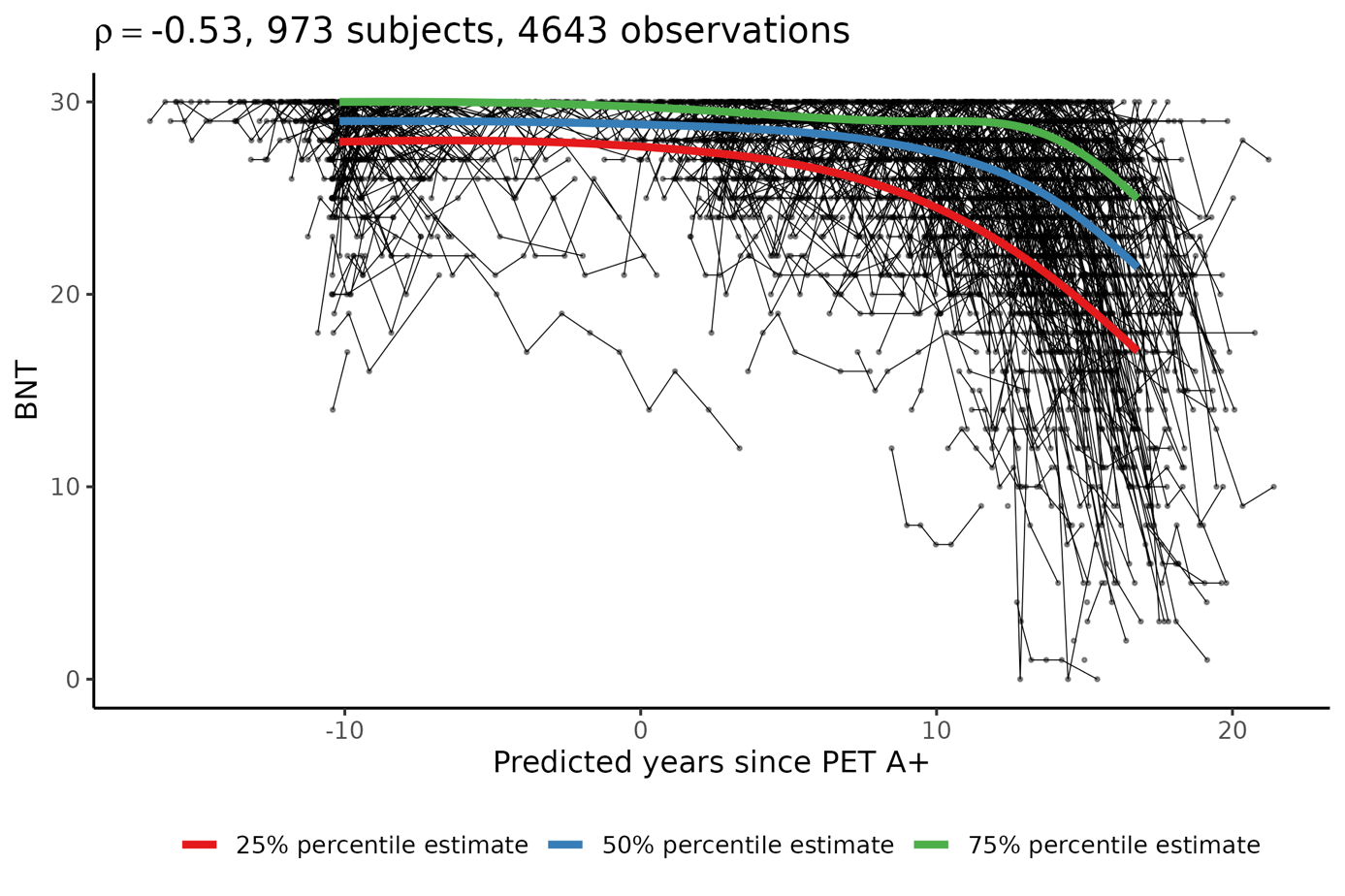


**Figure S13.** **Trajectories of Boston Naming Test (BNT30) across predicted disease time.** Observed longitudinal trajectories and quantile regression curves (25th, 50th, 75th percentiles) plotted against predicted years since amyloid PET positivity.


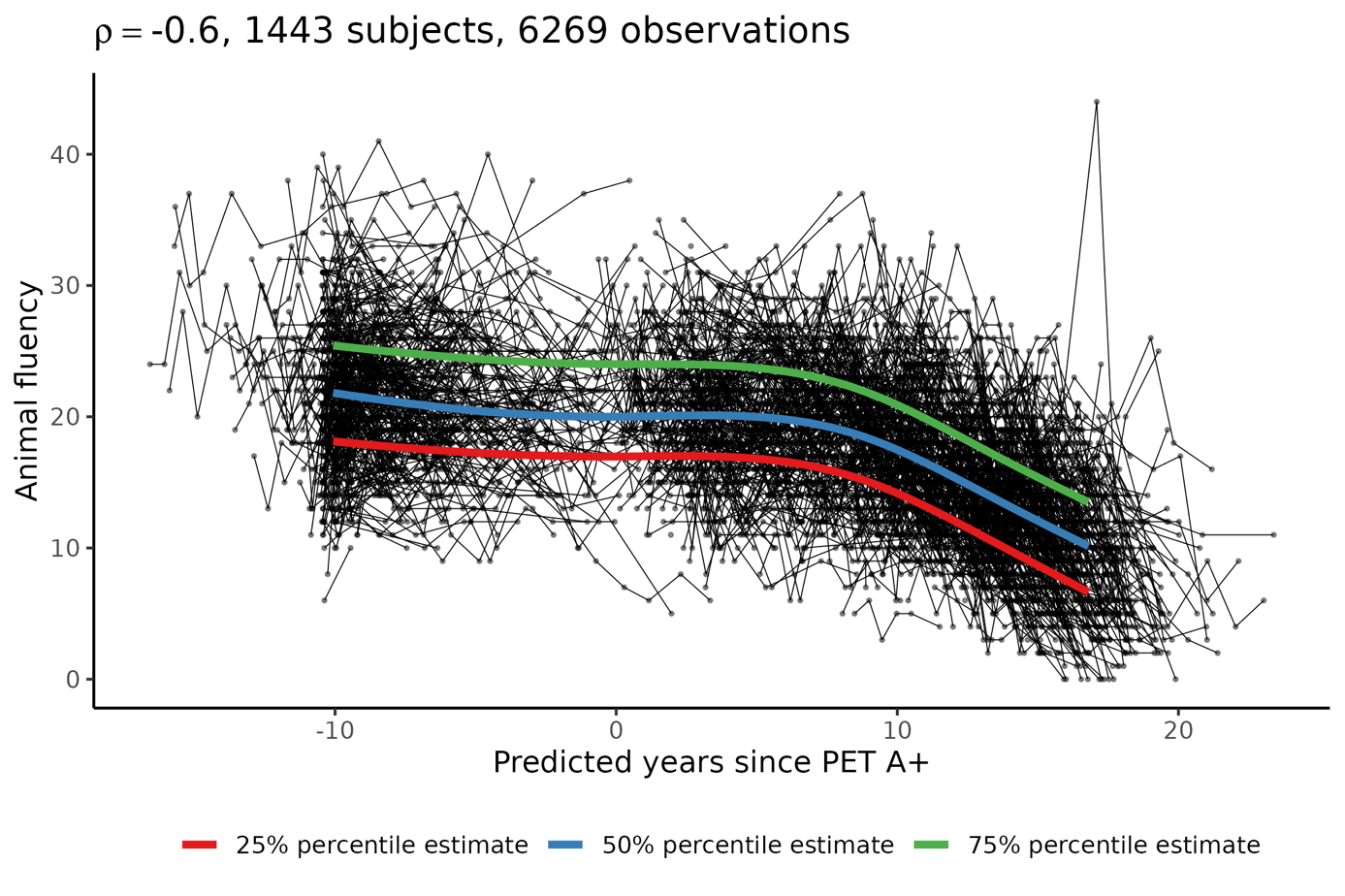


**Figure S14.** **Trajectories of Animal Fluency across predicted disease time.** Observed longitudinal trajectories and quantile regression curves (25th, 50th, 75th percentiles) plotted against predicted years since amyloid PET positivity.


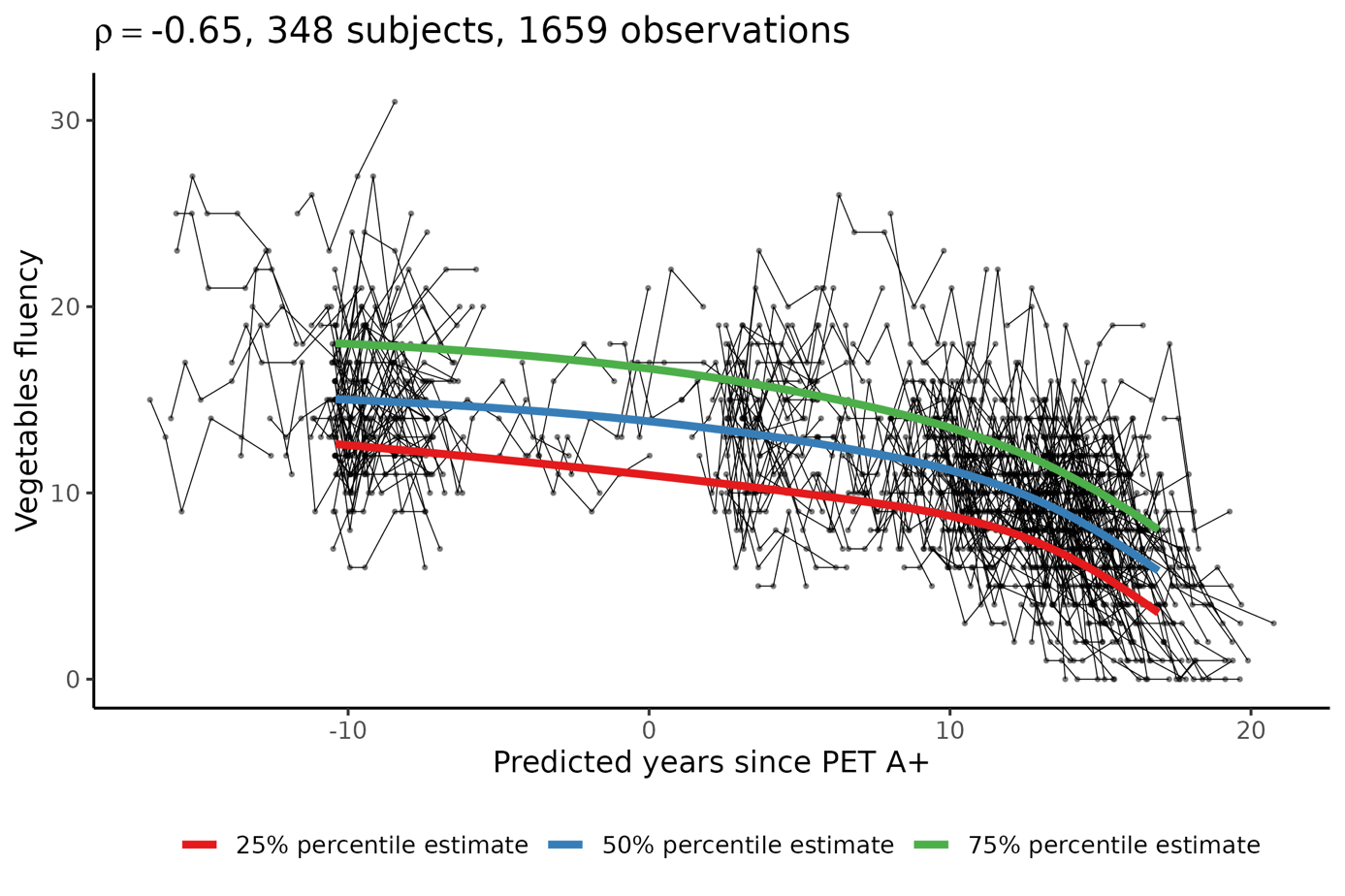


**Figure S15.** **Trajectories of Vegetables Fluency across predicted disease time.** Observed longitudinal trajectories and quantile regression curves (25th, 50th, 75th percentiles) plotted against predicted years since amyloid PET positivity.


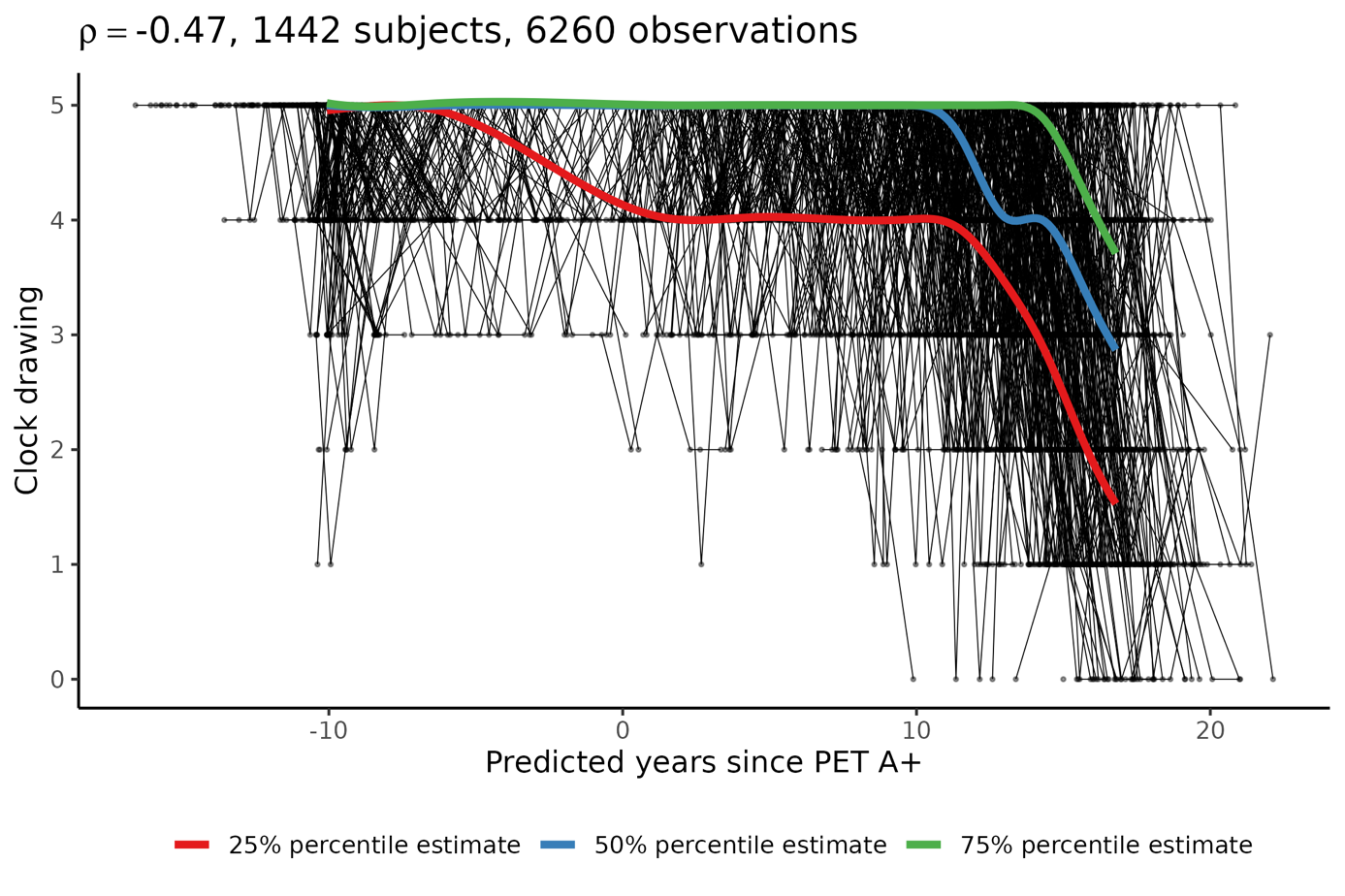


**Figure S16.** **Trajectories of Clock Drawing Test across predicted disease time.** Observed longitudinal trajectories and quantile regression curves (25th, 50th, 75th percentiles) plotted against predicted years since amyloid PET positivity.


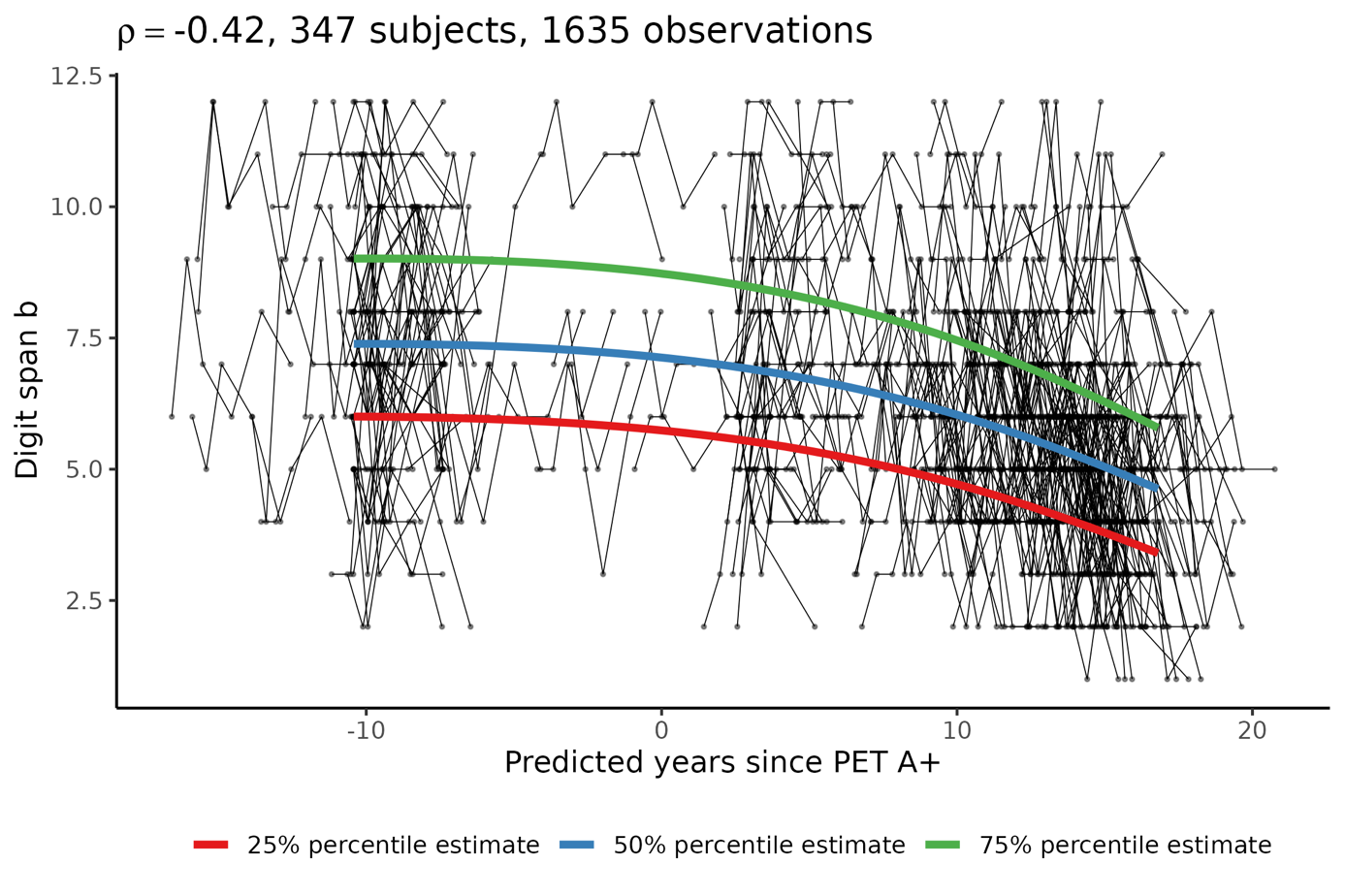


**Figure S17.** **Trajectories of Digit Span Backward (Digit Span b) across predicted disease time.** Observed longitudinal trajectories and quantile regression curves (25th, 50th, 75th percentiles) plotted against predicted years since amyloid PET positivity.


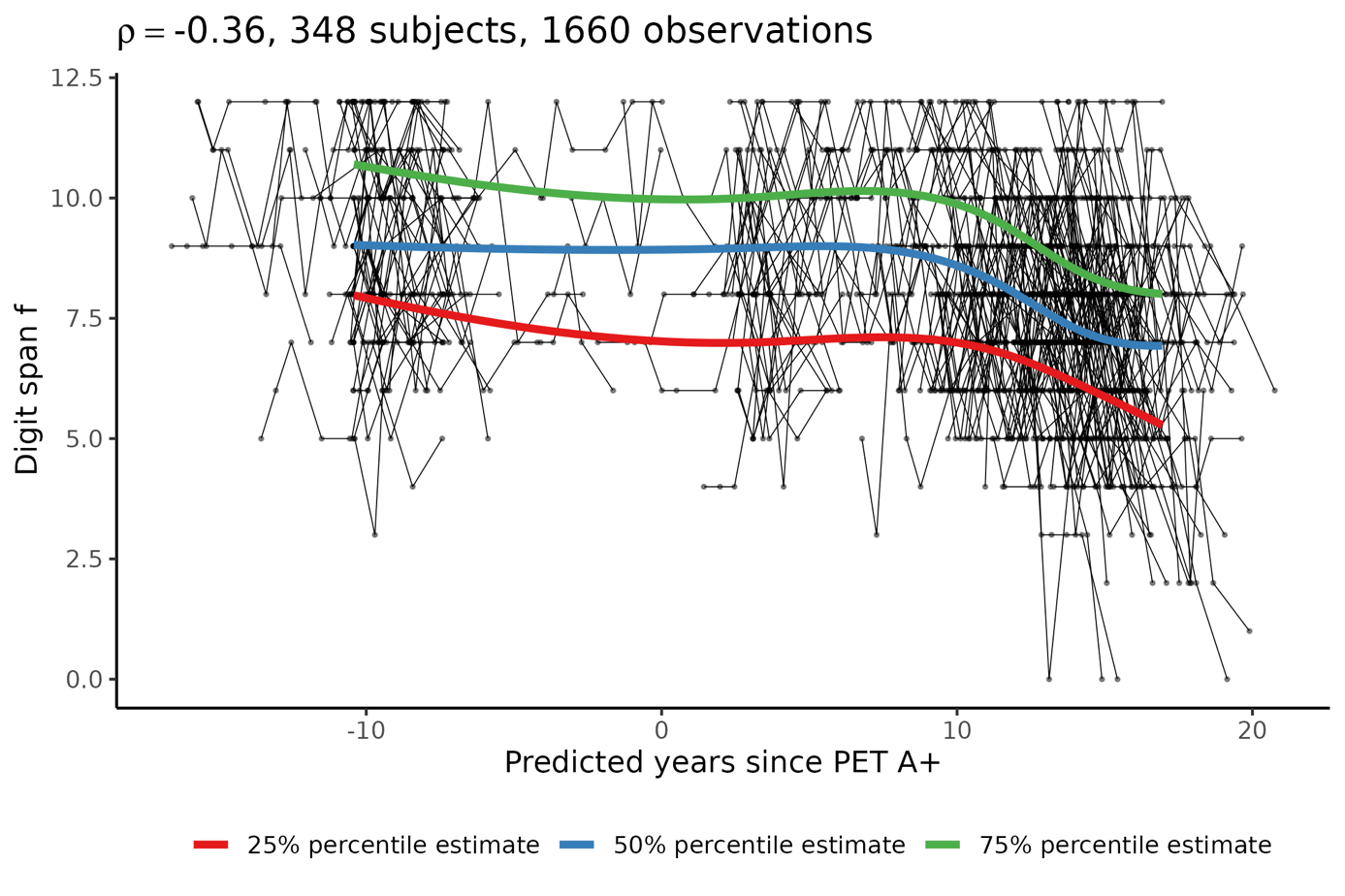


**Figure S18.** **Trajectories of Digit Span Forward (Digit Span f) across predicted disease time.** Observed longitudinal trajectories and quantile regression curves (25th, 50th, 75th percentiles) plotted against predicted years since amyloid PET positivity.


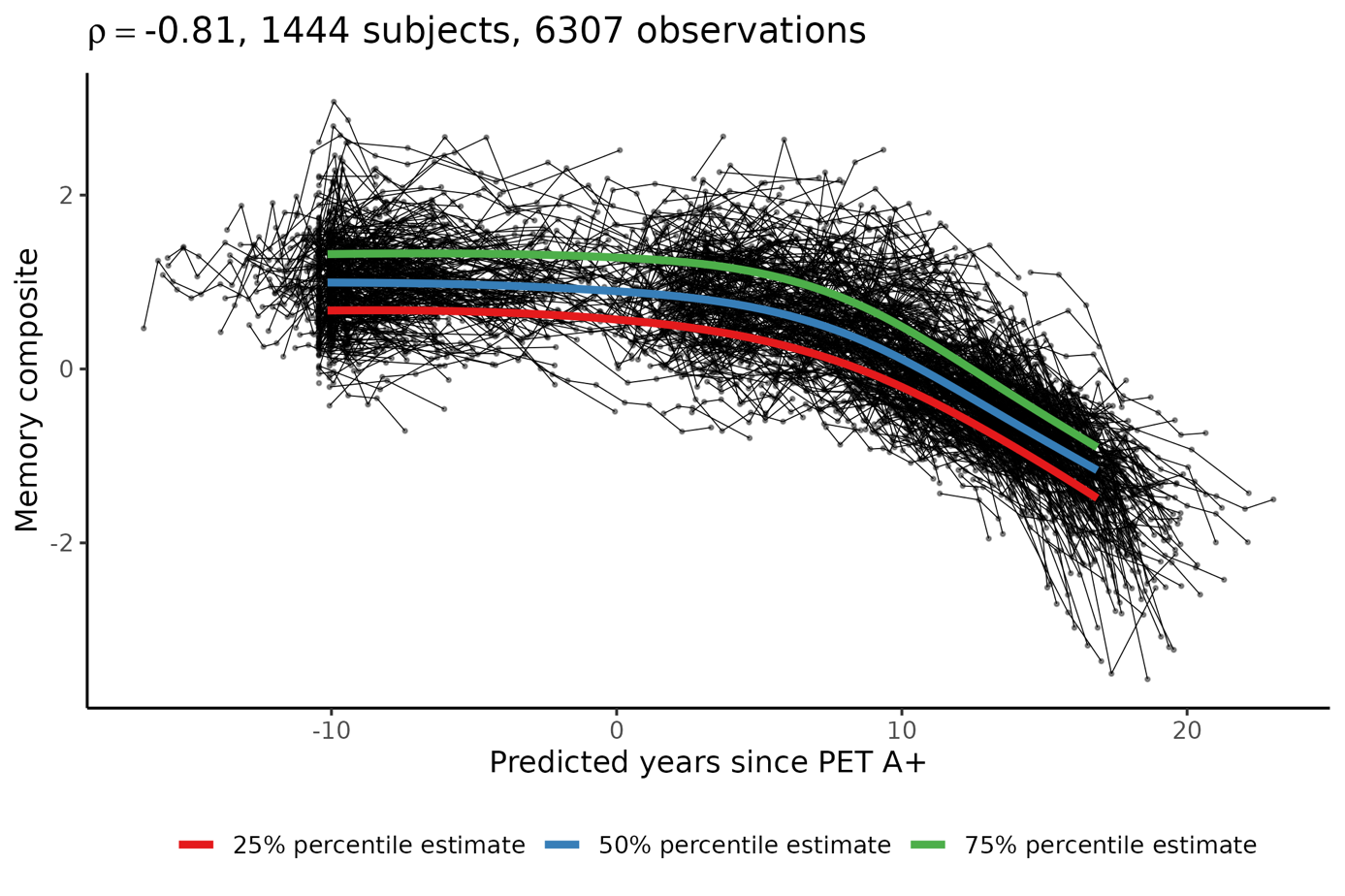


**Figure S19.** **Trajectories of Memory composite across predicted disease time.** Observed longitudinal trajectories and quantile regression curves (25th, 50th, 75th percentiles) plotted against predicted years since amyloid PET positivity.


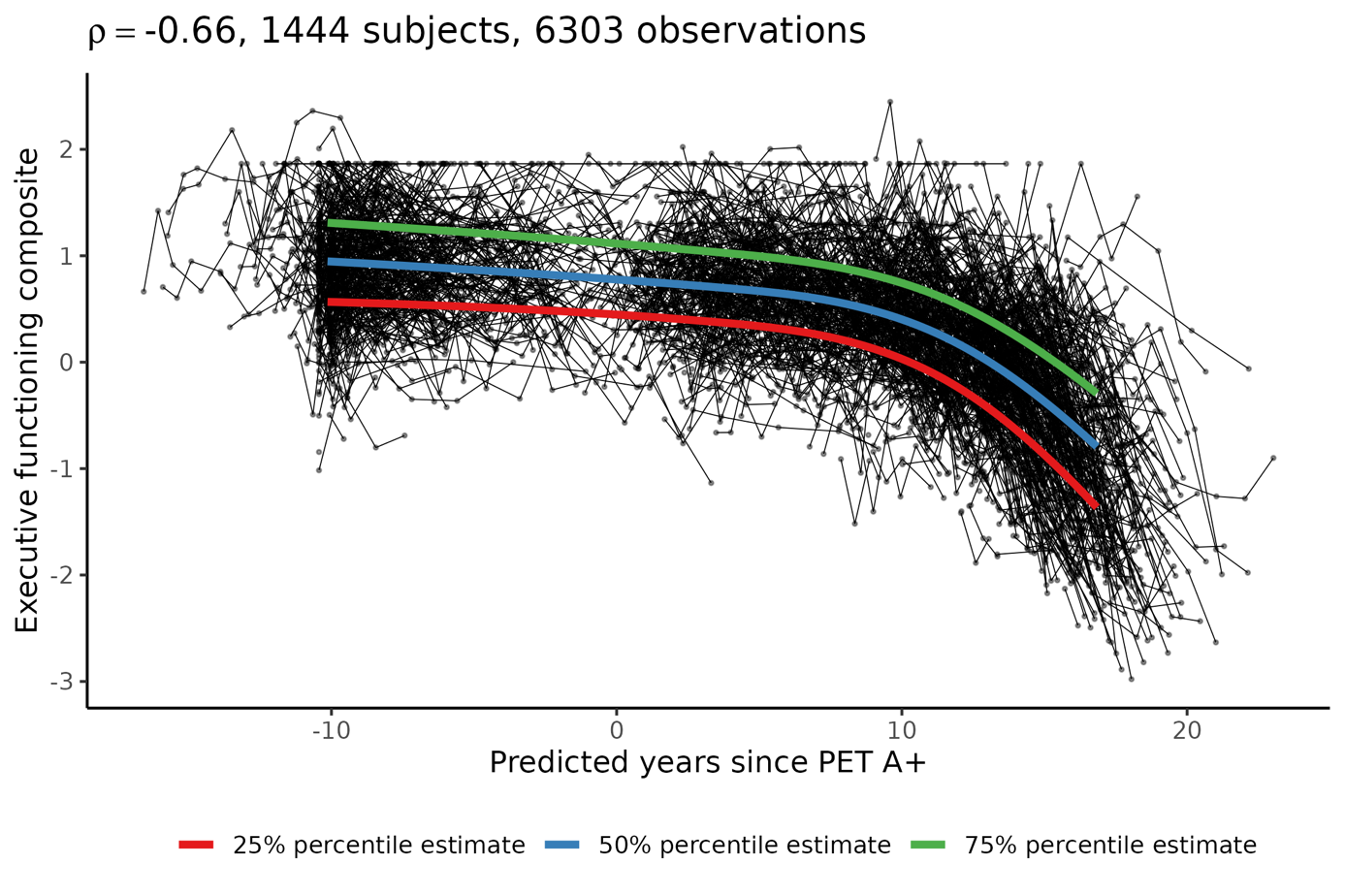


**Figure S20.** **Trajectories of Executive functioning composite across predicted disease time.** Observed longitudinal trajectories and quantile regression curves (25th, 50th, 75th percentiles) plotted against predicted years since amyloid PET positivity.


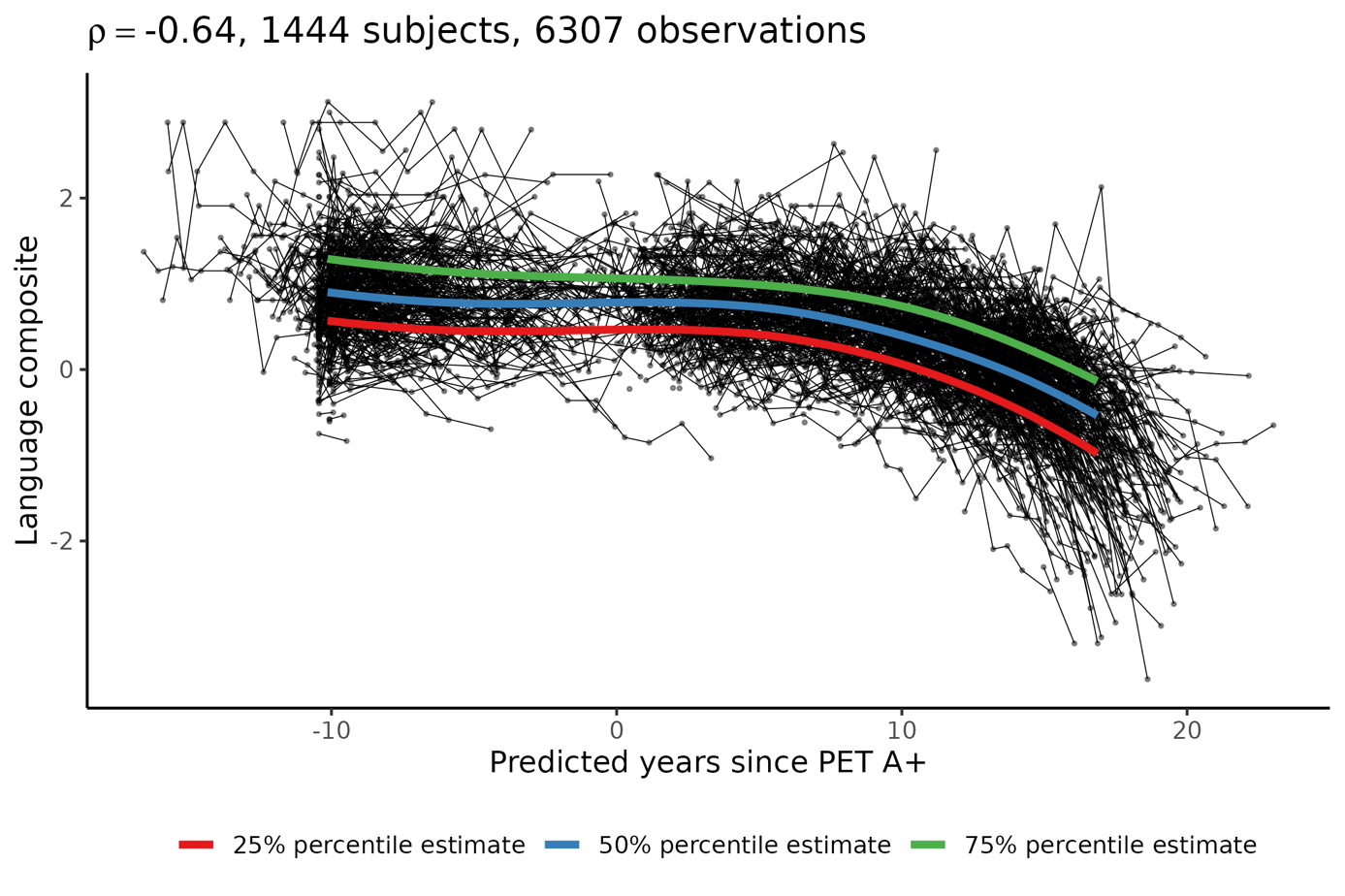


**Figure S21** **Trajectories of Language composite across predicted disease time.** Observed longitudinal trajectories and quantile regression curves (25th, 50th, 75th percentiles) plotted against predicted years since amyloid PET positivity.


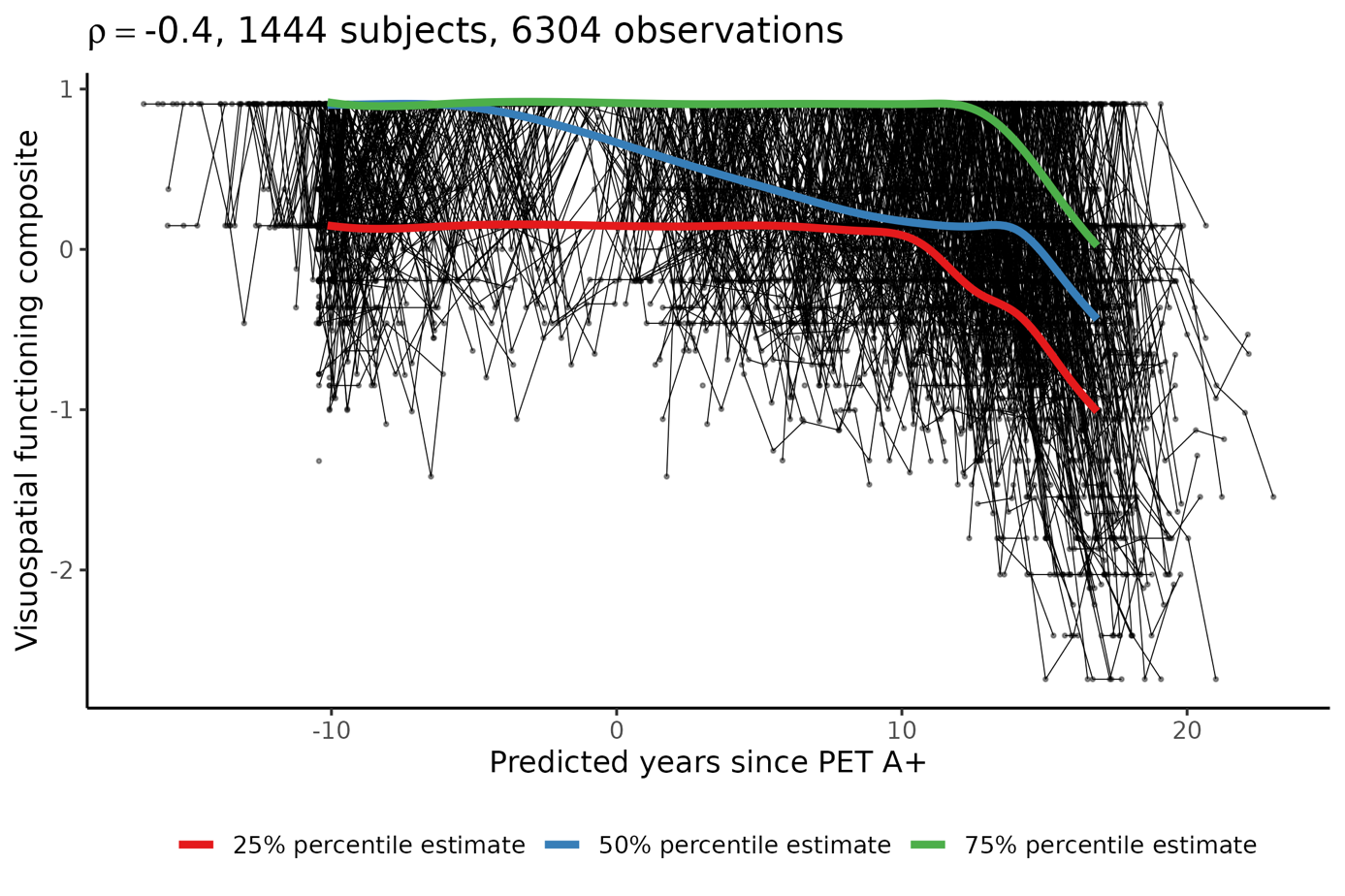


**Figure S22.** **Trajectories of Visuospatial composite across predicted disease time.** Observed longitudinal trajectories and quantile regression curves (25th, 50th, 75th percentiles) plotted against predicted years since amyloid PET positivity.
